# De novo design and bioactivity prediction of SARS-CoV-2 main protease inhibitors using recurrent neural network-based transfer learning

**DOI:** 10.1186/s13065-021-00737-2

**Published:** 2021-02-02

**Authors:** Marcos V. S. Santana, Floriano P. Silva-Jr

**Affiliations:** LaBECFar–Laboratório de Bioquímica Experimental e Computacional de Fármacos, Instituto Oswaldo Cruz, Fundação Oswaldo Cruz, Rio de Janeiro, RJ 21040-900 Brazil

**Keywords:** COVID-19, SARS-CoV-2, Transfer learning, De novo drug design, Generative model, Ulmfit

## Abstract

The global pandemic of coronavirus disease (COVID-19) caused by SARS-CoV-2 (severe acute respiratory syndrome coronavirus 2) created a rush to discover drug candidates. Despite the efforts, so far no vaccine or drug has been approved for treatment. Artificial intelligence offers solutions that could accelerate the discovery and optimization of new antivirals, especially in the current scenario dominated by the scarcity of compounds active against SARS-CoV-2. The main protease (M^pro^) of SARS-CoV-2 is an attractive target for drug discovery due to the absence in humans and the essential role in viral replication. In this work, we developed a deep learning platform for de novo design of putative inhibitors of SARS-CoV-2 main protease (M^pro^). Our methodology consists of 3 main steps: (1) training and validation of general chemistry-based generative model; (2) fine-tuning of the generative model for the chemical space of SARS-CoV- M^pro^ inhibitors and (3) training of a classifier for bioactivity prediction using transfer learning. The fine-tuned chemical model generated > 90% valid, diverse and novel (not present on the training set) structures. The generated molecules showed a good overlap with M^pro^ chemical space, displaying similar physicochemical properties and chemical structures. In addition, novel scaffolds were also generated, showing the potential to explore new chemical series. The classification model outperformed the baseline area under the precision-recall curve, showing it can be used for prediction. In addition, the model also outperformed the freely available model Chemprop on an external test set of fragments screened against SARS-CoV-2 Mpro, showing its potential to identify putative antivirals to tackle the COVID-19 pandemic. Finally, among the top-20 predicted hits, we identified nine hits via molecular docking displaying binding poses and interactions similar to experimentally validated inhibitors.

## Introduction

The global pandemic of coronavirus disease (COVID-19) caused by SARS-CoV-2 (severe acute respiratory syndrome coronavirus 2) created a rush to discover drug candidates against the virus [1–3]. As of June 2020, no vaccine or molecule has been approved for treatment of COVID-19, despite many molecules being screened and entering clinical trials, including remdesivir, chloroquine and lopinavir [4–6]. Therefore, there is an urge to boost drug discovery campaigns in order to identify safe and potent antivirals to tackle the COVID-19 pandemic. Moreover, the present efforts could form the basis of drug discovery strategies if a new coronavirus pandemic occurs.

Coronaviruses are enveloped, single-stranded RNA viruses members of the family *Coronaviridae *[7]. Their genome is approximately 30 kb and contains a variable number of open reading frames (ORFs) which encode 16 nonstructural (nsp), 4 structural and several accessory proteins [8–12]. ORF1a/b translates to two polyproteins, pp1a and pp1ab, which are processed by two proteases into structural and nonstructural proteins [13–15]. In SARS-CoV-2 the nonstructural protein 5 (nsp 5) is the main protease and is essential for viral replication [2, 16].

The main protease 3-chymotrypsin-like (M^pro^ or 3C-like) of SARS-CoV-2 is a cysteine protease and consists of a homodimer organized in three domains (I–III) [17]. The active site is located on the cleft between domains I and II and features the catalytic dyad Cys-His [2, 17]. M^pro^ is conserved among coronaviruses, sharing ~ 76% sequence similarity with SAR-CoV-1 M^pro^, and there are no homologs in humans, making it an attractive target for drug discovery [2, 7, 18]. Furthermore, the high sequence similarity to SARS-CoV-1 M^pro^ suggests that previously described inhibitors could be used as templates to design new inhibitors to boost the drug arsenal against SARS-CoV-2.

The development of treatments against SARS-CoV-2 is a fast-changing field and a comprehensive review is out of the scope of this work. However, we instruct the interested reader to a recent review from our group about COVID-19 molecular targets and drug repurposing strategies [19] as well as other reviews about treatment options [20–22]. Due to the lack of antivirals targeting SARS-CoV-2, computational approaches could offer fast solutions to design, prioritize and optimize small molecules for screening. In this scenario, artificial intelligence (AI) has been extensively used to explore the chemical and biological space in large molecular databases to find drugs that could be repurposed and novel antiviral activities [10, 23–27].

In this work we used ULMFiT [28] to train a chemistry model to generate molecules in the same chemical space as molecules screened against SARS-CoV main protease (M^pro^); and a classification model to predict the bioactivity of the generated molecules on SARS-CoV-2 M^pro^. The molecules predicted as active were further analysed using molecular docking to investigate possible interactions with M^pro^.

## Methods

### Dataset and molecule representation

We used ChEMBL 26 [29] and PubChem [30] as sources of chemical data in the format of SMILES (*Simplified Molecular Input Line Entry Specification*) strings [31]. We downloaded 1,940,733 small molecules from ChEMBL and submitted them to standardization to neutralize charges, remove salts, normalization of groups and converting the SMILES to the canonical form. The data was filtered to keep only molecules with atoms in the set *L* = {H, C, N, O, P, S, Br, I, Cl, F}. We also removed molecules with less than 10 or more than 50 heavy atoms. The filtering and standardization steps were implemented using RDKit 2020.01.1 (https://www.rdkit.org/).

For fine-tuning, the dataset consisted of over 280 K molecules screened against SARS-CoV-1 M^pro^ available on PubChem (AID: 1706). Originally, AID1706 consisted of 405 active molecules, but we augmented it with 224 inhibitors collected from literature by Tang et al., (https://github.com/tbwxmu/2019-nCov) [32]. In total, our fine-tuning dataset was highly unbalanced, with 629 active molecules and 288,940 inactive ones. Molecules in the fine-tuning dataset were submitted to the same preprocessing protocol described above.

**Fig. 1:**
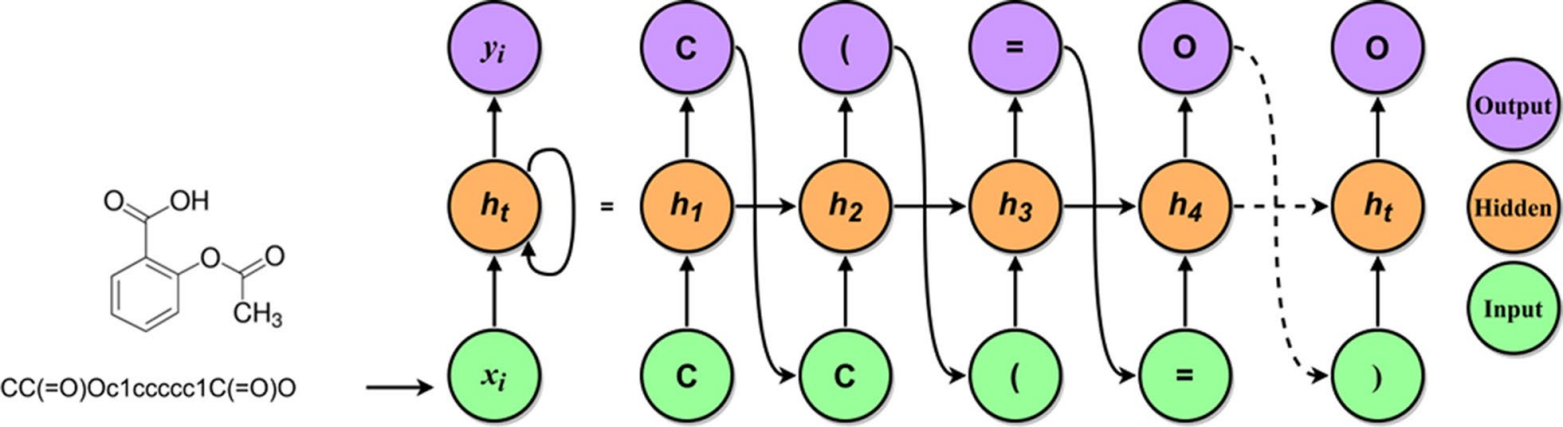
Overview of the basic concepts showing an unfolded recurrent neural network. Molecules are represented as SMILES strings in order to train a chemical model. The SMILES string is split into individual tokens representing atoms and special environments (e.g., charged groups and stereochemistry). The tokenized molecule is then used as input to a recurrent neural network (RNN). At each time step *t,* the model receives as input a token and the hidden state of the previous step (*h*_*t−1*_). It then updates its own hidden state *h*_*t*_*,* and outputs the next token in the sequence (*y*_*i*_)

In this work, we used molecules represented as SMILES strings as input to the model (Fig. 1). Each SMILES string is a one-line textual representation of a molecule, where each atom is represented by its atomic symbol (e.g., C, for carbon; N for nitrogen etc.).

In order to use molecules as SMILES strings as input, the SMILES were initially split into individual characters or tokens, representing the individual atoms in the molecule and special chemical environments, (e.g., [OH−] and stereochemistry information). After tokenization, we used a string-to-integer dictionary to convert each token to a unique integer. In total, the dictionary consisted of N entries, including beginning of string (BOS), end of string (EOS) to represent the start and end of each SMILES string, respectively. We also added padding tokens (needed for the classification task) and UNK tokens to deal with tokens that were not covered by the dictionary, which could be useful when dealing with molecules with exotic groups. To summarize, each molecule was represented by an integer array, where each number represented an atom or chemical environment.

### Model architecture

**Fig. 2:**
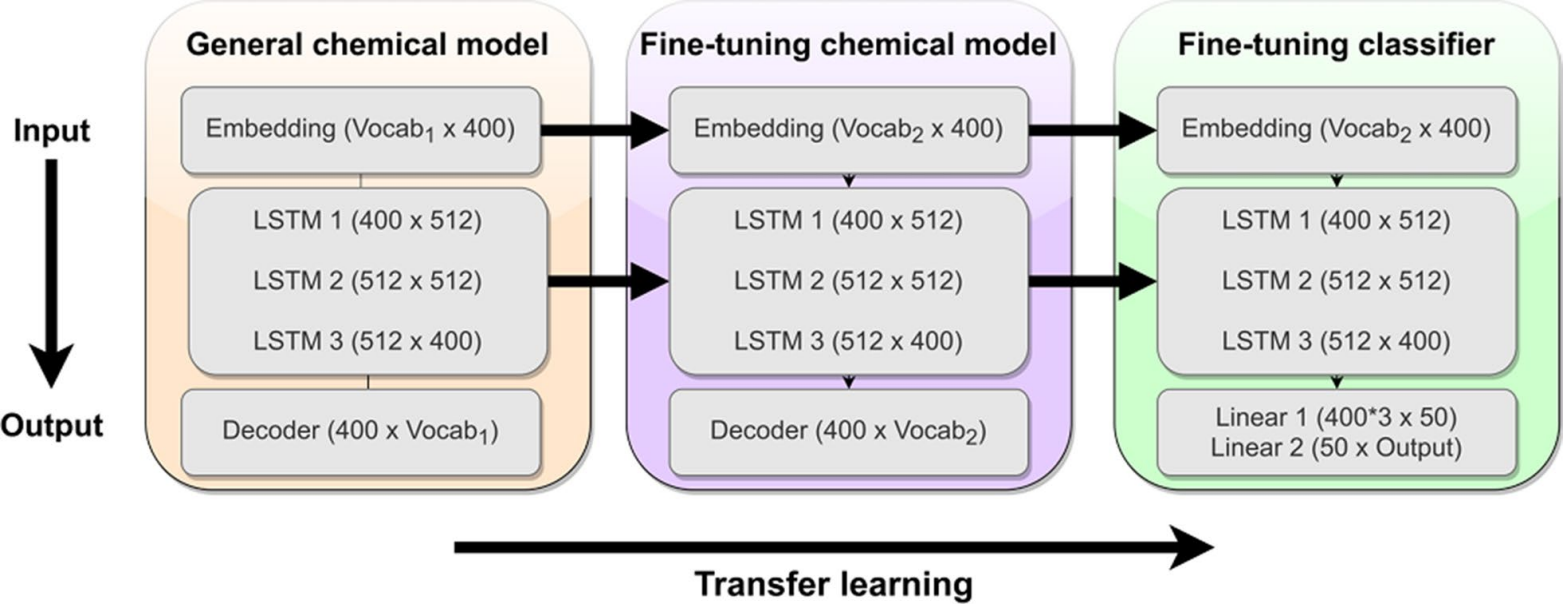
Overview of the ULMFit approach. Initially, a general chemical model is trained to learn the “chemical language” contained in a collection of input molecules. The learned features can then be transferred to a target-task and adapted to the idiosyncrasies of the data. These “chemical models” can be used to generate molecules on demand. The last step consists of using the fine-tuned features to train a classifier that predicts bioactivity

We used AWD-LSTM as a base architecture [33], which is a kind of recurrent neural network (RNN) that can handle sequential data and learn short and long-term dependencies between items in a sequence [34]. The architecture consists of an embedding layer, three LSTM (Long-Short Term Memory) layers and a fully connected linear layer (Fig. 2). Similar to the original ULMFit method [28] (see Additional file 1: Part I), we used an embedding layer with shape N × 400, where N is the number of input tokens in our dictionary and 400 the number of outputs.

We initially trained the model using the default 1152 hidden units of ULMFit. However, the total training time was superior to the GPU time available. Therefore, we changed the number of hidden units to 512 while still maintaining performance. During training, the embedding layer receives the inputs and maps them to a latent feature space that contains the contextualized information about a molecule, which can be learned. The embedding and LSTM layers are the encoder of the model, responsible for learning the “chemical language” and short and long-term dependencies between each token. The final layer was the decoder and consisted of a linear layer with a softmax activation that outputs probabilities for each predicted token.

For fine-tuning the classifier, we used the same AWD-LSTM architecture as the language model but augmented it with two additional linear blocks with relu activations. In other words, only the linear blocks of the classifier were trained from scratch, which makes the ULMFit approach very flexible in the quantitative structure–activity relationship (QSAR) context, since the chemical language learned by the general model can be reused [35].

The input to the classifier is the activation of the last time step **h**_**t**_ of the encoder concatenated with the max-pooled and mean-pooled activations of previous time steps. This pooling operation returns the maximum and average activations of previous time steps, allowing the model to focus on the most important features to make a prediction [28]. In addition, batch normalization and dropout layers were used between each layer to avoid overfitting. The final layer consisted of a linear layer with softmax function to output the probabilities for bioactivity prediction, classifying each molecule as “Active” or “Inactive”.

### Training

We trained the general chemical model from scratch for 10 epochs using a constant learning rate of 3 × 10^–3^. We randomly selected 10% of the data as a validation set to monitor the performance and avoid overfitting. For fine-tuning, we started with the pretrained model and fine-tuned it using discriminative learning rates and gradual unfreezing as proposed by Howard & Ruder in the original ULMFit paper [28]. In this context, since each layer captures different types of information [36], it is sensible to fine-tune each layer with a different learning rate [28]. The learning rate was adjusted using the function η^layer−1^ = η^layer^/2.6, used in the original ULMFiT approach, where η is the learning rate and layer is the number of a specific layer.

Training with gradual unfreeze initially trains only the linear blocks of the classifier, while keeping the parameters of the encoder frozen. We initially trained the classifier for 4 epochs and then unfroze and fine-tuned each layer every 3 epochs until convergence and all layers were fine-tuned [37]. This method of training slowly adapts the classifier to the new task and minimizes the risk of catastrophic forgetting that could happen when fine-tuning all layers at once [37].

### Implementation

We implemented our model using Fastai v1 library [37] (https://docs.fast.ai). The codes and models for reproducibility are freely available on request. All codes were written in Python 3 and ran on *Google Colaboratory* (Colab) (Google, 2018) using Ubuntu 17.10 64 bits, with 2.3 GHz cores and e 13 GB RAM, equipped with NVIDIA Tesla K80 GPU with 12 GB RAM.

### Validation of the generative model

To validate the general and fine-tuned chemical models, we computed the number of novel, unique and valid molecules generated. We define these metrics as follows:

- Validity: percentage of chemically valid SMILES generated by the model according to RDKit. A SMILES string is considered valid if it can be parsed by RDKit without errors;
- Novelty: percentage of valid molecules not present in the training set;
- Uniqueness: percentage of unique canonical SMILES generated.

The SMILES strings were generated by inputting the start token “BOS” and progressed until the end token “EOS” token was sampled or a predefined size was reached. The probability for each predicted token was calculated with the output of the softmax function and adjusted with the hyperparameter temperature (T). The sampling temperature is a hyperparameter that adjusts the output probabilities for the predicted tokens and controls the degree of randomness of the generated SMILES and the confidence of predicting the next token in a sequence [38]. Lower temperatures make the model more conservative and output only the most probable token, while higher temperatures decrease the confidence of predictions and make each token equally probable [39, 40]. The probability of predicting the *i*-th token is calculated as (Eq. 1):

1 $$p_{i} \; = \;\frac{{e^{{(y_{i} /T)}} }}{{\sum\nolimits_{j\; = \;1}^{k} {e^{{(y_{i} /T)}} } }}$$

where *y*_*i*_ is the softmax output, *T* is the temperature and *j* ranges from *i* to *K* number of maximum tokens to sample from the model.

### Validation of the classifier

The classifier performance was evaluated with fivefold cross-validation. We performed two types of splitting: (1) random split into training, validation and test sets using a 80:10:10 ratio, and (2) Scaffold-based splitting in order to ensure that the same scaffolds were not present in training and validation sets. In addition, a dataset of 880 fragments screened against SARS-CoV-2 M^pro^ using X-ray crystallography was used as an external evaluation set (https://www.diamond.ac.uk/covid-19/for-scientists/Main-protease-structure-and-XChem/Downloads.html). Since the dataset was highly unbalanced, we used the area under the precision-recall curve (AUC-PR) as the key metric to evaluate the performance, which is more informative in this scenario [41]. The AUC-PR can be calculated from a plot of precision X recall (or sensitivity):

2 $$Se\; = \;\frac{TP}{{TP\; + \;FN}}$$

3 $$Sp\; = \;\frac{TN}{{TN\; + \;FP}}$$

4 $$Pre\; = \;\frac{TP}{{TP\; + \;FP}}$$

where TP, TN, FP and FN are the numbers of true positives, true negatives, false positives and false negatives, respectively.

We also compared the performance of our classifier on the external test set with Chemprop, a freely available message passing neural network (MPNN) that has been used to repurpose drugs to SARS-CoV-2 (http://chemprop.csail.mit.edu/predict) [42].

### Chemical space analysis

We evaluated the chemical space coverage by computing Uniform Manifold Approximation and Projection (UMAP) plots of Morgan circular fingerprints or Extended Connectivity Fingerprints (ECFP) of length 1,024 bits and radius of 2 bonds. UMAP is a dimensionality reduction method used to visualize high-dimensional data (in this case ECFP4 fingerprints) in just 2 dimensions (2D). Using this method, similar molecules are clustered close to each other, while also preserving the global structure of the original high-dimensional data [43]. In addition, we investigated the Tanimoto similarity between the generated molecules and true inhibitors in terms of structure and Bemis-Murcko scaffolds [44].

### Docking protocol

A molecular docking simulation was carried out using the Protein–Ligand Ant System (PLANTS) v.1.2 docking software. The active site Cys145 was used as the center of the simulation box and 15Å were added to each cartesian direction, in order to include the S1/1′, S2 and S3 subsites of M^pro^ active site. The molecules were scored with the ChemPLP scoring function, using the default search speed parameter of PLANTS v1.2 (search speed = 1). All docking calculations were carried out using the crystal structure of SAR-CoV-2 M^pro^ (PDB: 6W79, resolution = 1.46 Å). Before submitting our molecule library to docking, we performed a round of redocking in order to validate our protocol. The results are shown in the Additional file 1: Part III: Molecular Docking.

### Physicochemical filtering and PAINS detection

In order to prioritize compounds for purchase and synthesis, we submitted the top 20 predicted hits to a pan assay interference compounds (PAINS) filter to remove potentially problematic molecules from our final selection. All calculations were carried out on the FAF-DRUGS4 server [45].

## Results and discussion

### General chemical model validation

We initially validated the chemical model trained on ChEMBL to access its potential to generate molecules using SMILES strings. The main metrics have been used to validate generative models in other works [39, 40, 46].

#### Validity, uniqueness and novelty of the generated molecules

We initially investigated the performance of different sampling temperatures on the proportion of valid, unique and novel molecules generated. Our results are summarized in Additional file 1: Part II, Table S1.

Overall, our results indicate that the general model can generate diverse and novel molecules (Additional file 1: Table S1). When sampling with *T* = 0.8, we obtained a good compromise of validity (98.73 ± 0.15%), uniqueness (98.69 ± 0.17%) and novelty (86.57 ± 0.36%) scores (Additional file 1: Table S1). Therefore, we decided to use *T* = 0.8 for the subsequent experiments. Most structural errors were associated with incomplete ring systems, where RDKit could not find matching pairs of brackets on the SMILES string, and a smaller proportion consisted of invalid valances, such as C^+5^ and Cl^+2^.

The performance of the general chemical model is in accordance with previous findings for LSTM-based models, with high validity, diversity and novelty scores [38–40, 46]⁠. For instance, Brown et al. benchmarked different generative methods, to access their potential in de novo drug design. Their LSTM model achieved validity, diversity and novelty scores higher than 90%, even higher than other machine learning methods, including variational autoencoders (VAE), generative adversarial networks (GAN’s), adversarial autoencoders (AAE) [46]⁠. In another study, Merk et al., pre-trained a model on 550 thousand SMILES from bioactive molecules from ChEMBL and then used it to generate target-specific inhibitors for peroxisome proliferator-activated receptor gamma (PPARγ) and trypsin, achieving novelty scores of 88% and 91%, respectively [47]. We also highlight the study by Moret et al., that adopted a similar approach to ours by using transfer learning and a LSTM model on low-data problems. Their model achieved high proportions of valid, unique and novel molecules (> 90%), which was further improved when data augmentation was used to increase the training size by including different representations of the same SMILES string [39].

#### Chemical space analysis

**Fig. 3:**
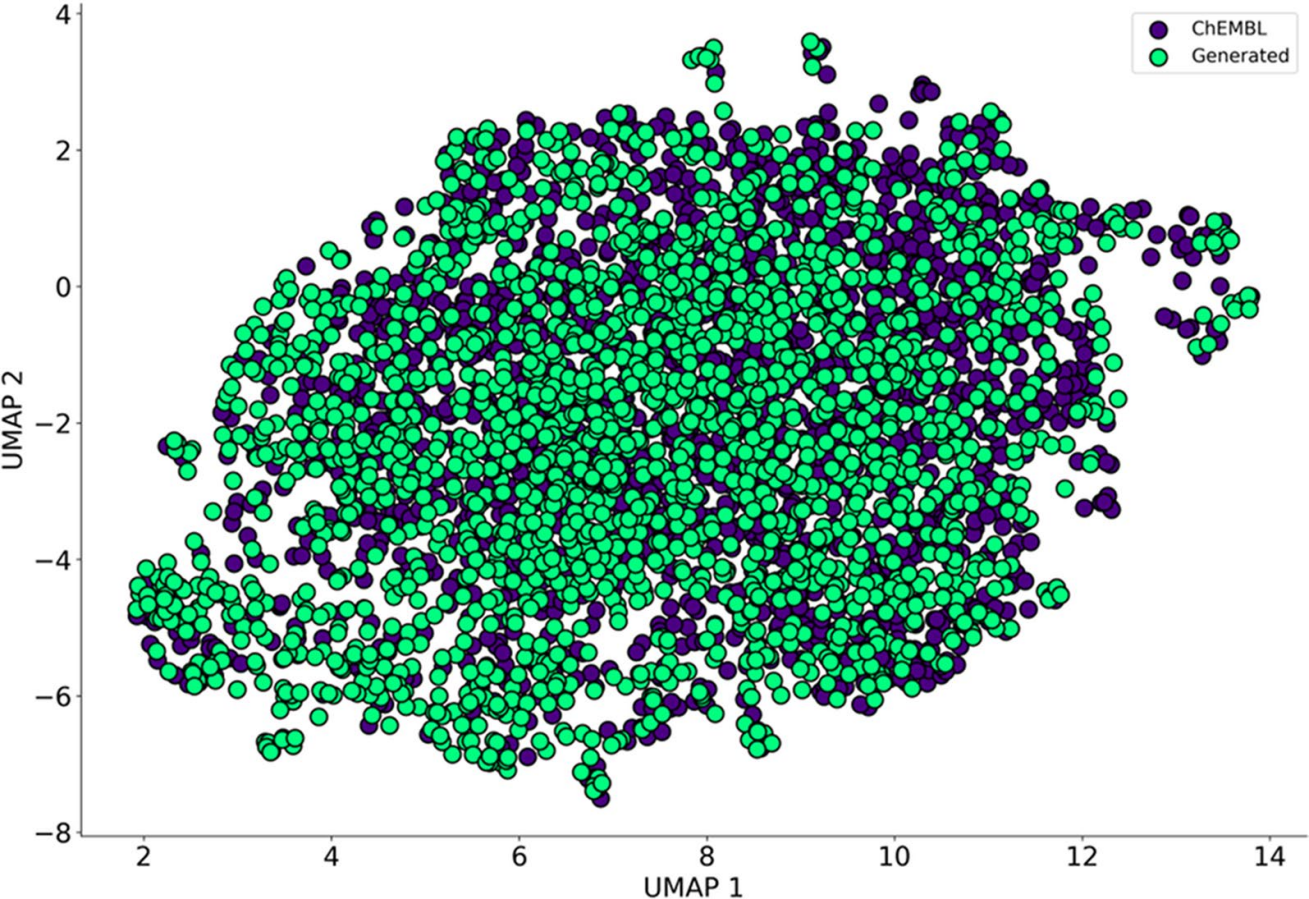
UMAP plot of the chemical space of molecules generated by the general chemical model and ChEMBL (2000 molecules were randomly selected for each set)

As shown in Fig. 3, the chemical spaces of ChEMBL and of the generated molecules have a high degree of overlap, indicating that the model captured the structural features from ChEMBL.

#### Scaffold diversity and novelty

**Fig. 4:**
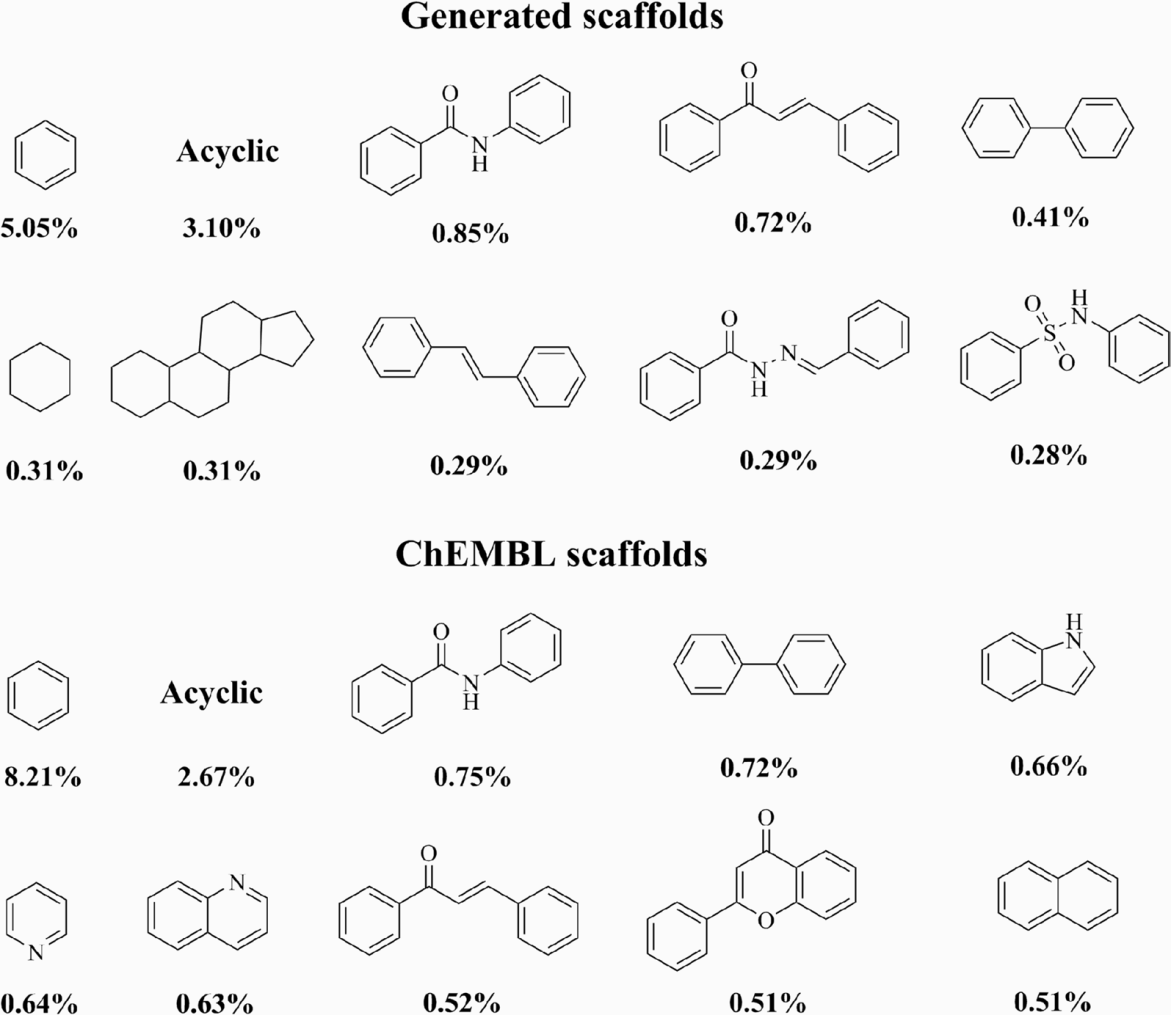
Top-10 most common scaffolds from the generated molecules and ChEMBL sets. The prevalence of each scaffold is also shown

We also investigated the chemical space of the generated scaffolds. For this task, we sampled 10,000 valid SMILES, representing 7538 unique Bemis-Murcko scaffolds (75.58%). The top-10 most common scaffolds were relatively simple, fragment-sized, with less than 30 heavy atoms and consisting of at most two 6-membered rings (Fig. 4). In addition, five of the top-10 most common scaffolds were also among the most common ChEMBL scaffolds, further demonstrating a relative overlap of chemical spaces (Fig. 4).

**Fig. 5:**
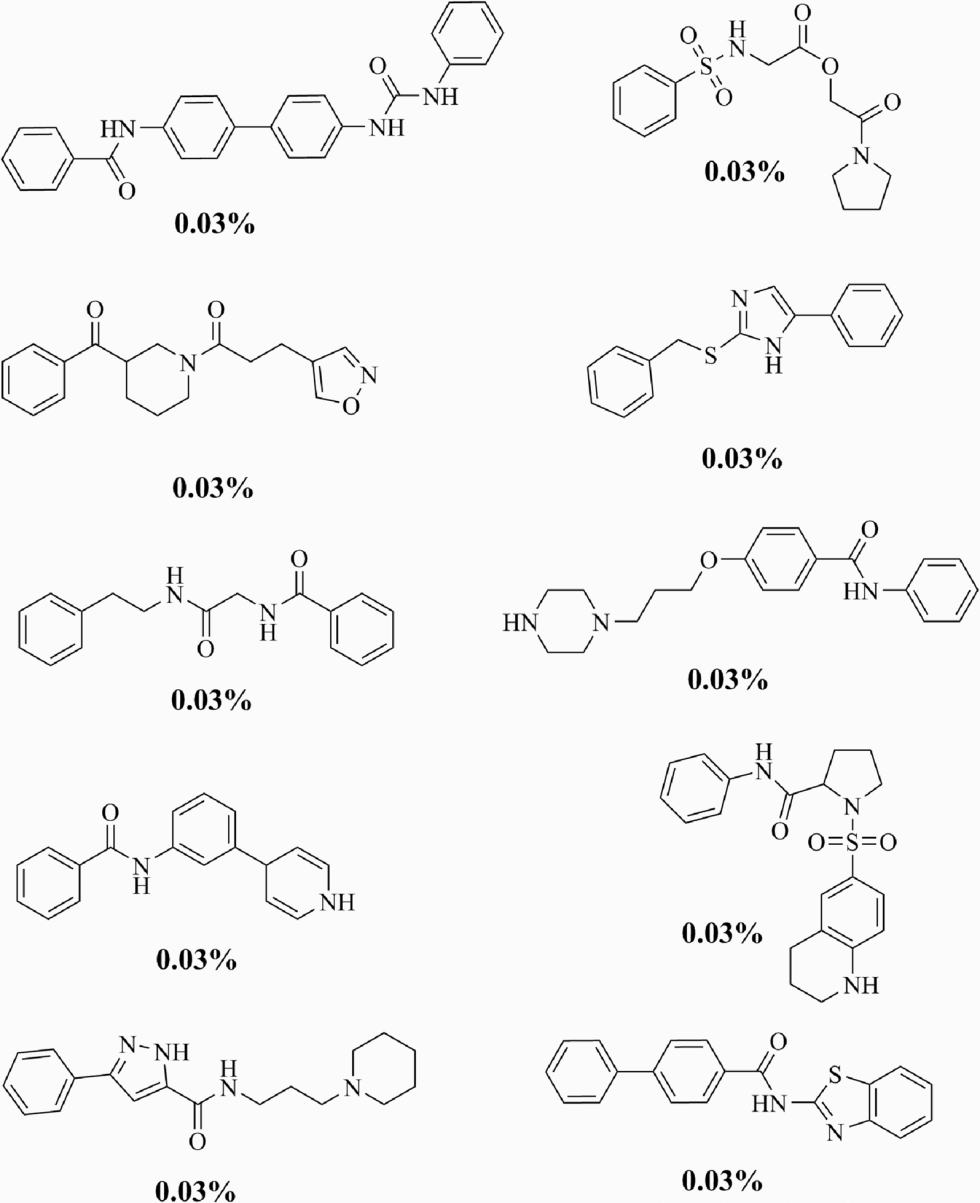
Top-10 most common novel scaffolds. The prevalence of each scaffold among the generated molecules is also shown

In terms of novelty, 3291 (43.66%) of the scaffolds were not present on the training data. In general, the frequencies of each novel scaffold in the generated set were low; each representing only 0.03% of all scaffolds (Fig. 5). Structurally, the novel scaffolds were more complex than the most common ones, with a higher number of heavy atoms and heterocycles.

The UMAP shows the scaffolds of 2,000 randomly selected molecules from ChEMBL and the generated set (Additional file 1: Part II, Figure S1). The plot highlights the overlap in chemical space of the scaffolds and corroborates our previous analysis that the LSTM model captured the chemical information from the training set.

### Fine-tuning for M^pro^ chemical space

We previously demonstrated that our generative model was able to generate valid, diverse and novel molecules and scaffolds. In the following experiments, the encoder of the LSTM model was used to fine-tune another model to generate a focused library for compounds active on SARS-CoV-1 M^pro^. The dataset of M^pro^ inhibitors was very small, with 629 active molecules and more than 280 K inactive ones. Therefore, a model that could conserve scaffolds associated with high activity and expand the chemical space would be a valuable tool to tackle the lack of chemical matter for the current and future coronaviruses pandemics.

#### Sampling temperature

Like our previous analysis, we initially evaluated the optimal temperature by sampling 2000 SMILES in five independent runs (10,000 in total). As expected, with *T* = 0.20 all molecules were valid due to the model only returning high confidence predictions about the next character in the SMILES string (Table 1). However, the generated molecules showed low uniqueness and novelty scores, indicating that the model is generating the same molecules at every round. Sampling with temperatures higher than 0.5 yielded high proportions of unique, valid and novel molecules (Table 1).

**Table 1:** Validity, uniqueness and novelty (mean ± std) of SMILES generated after training

Temperature	Validity (%)	Uniqueness (%)	Novelty (%)
0.20	100.00 ± 0.00	39.79 ± 0.27	33.21 ± 0.59
0.50	99.98 ± 0.03	99.05 ± 0.30	78.44 ± 0.78
0.60	99.95 ± 0.04	99.05 ± 0.18	81.80 ± 1.19
0.70	99.80 ± 0.10	99.58 ± 0.16	85.10 ± 0.58
0.75	99.72 ± 0.15	99.58 ± 0.12	85.85 ± 0.68
0.80	99.44 ± 0.21	99.36 ± 0.20	87.11 ± 0.59
1.00	97.21 ± 0.39	97.15 ± 0.15	88.66 ± 0.95
1.20	89.95 ± 0.23	89.84 ± 0.24	85.38 ± 0.87

We sampled 2000 SMILES for each temperature in five independent runs (10,000 in total)

#### Optimal SMILES size

**Fig. 6:**
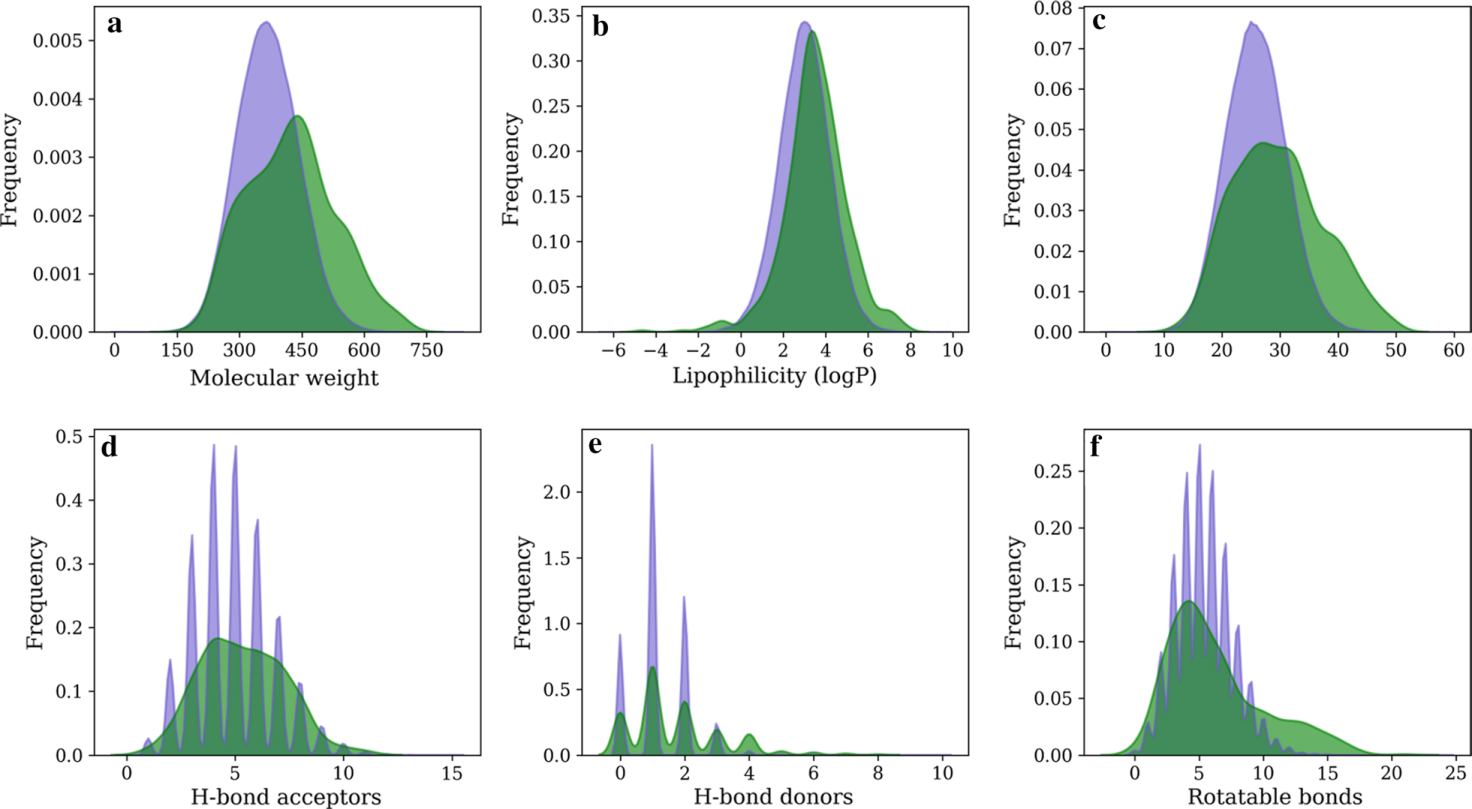
Physicochemical properties for the generated molecules (purple) and known SARS-CoV-1 M^pro^ inhibitors (green). **a** Molecular weight; **b** logP; **c** heavy atom count; **d** H-bond acceptors; **e** H-bond donors; and **f** number of rotatable bonds

We also investigated the distribution of molecular weights as a function of the maximum size of the SMILES strings the model could generate. Figure 6 shows that the model can generate a range of structures, from fragments (SMILES size in the range 10–30 characters) to molecules with molecular weights > 500 Da, outside the ranges of classical drug-like physicochemical filters, such as Lipinski’s rule of 5 (molecular weight < 500 Da, HBA < 10, HBD < 5 and logP < 5) [48]. The average molecular weight of the generated molecules stabilized between 350 and 400 Da when the maximum number of characters per SMILES string was higher than 60. This flexibility to generate molecules with different sizes allows our model to be used in different virtual screening settings, from fragments to lead / drug -like campaigns. For the next analysis, we generated 70,000 valid SMILES using the fine-tuned model setting *T* = 0.80 and the maximum size of SMILES set to 50, in order to generate molecules in the drug-like chemical space.

### Generating M^pro^-focused compound libraries

Having set the sampling temperature, we generated 70,000 valid SMILES using the fine-tuned model. After removing duplicates, we obtained 67,527 unique molecules (96.47%). The generated molecules displayed a slight shift to lower values of molecular weight (MW), logP and number of heavy atoms, indicating that, in general, the model generated smaller and more hydrophilic molecules compared to M^pro^ inhibitors (Fig. 6a–c). On the other hand, the distributions of the number of rotatable bonds, H-bonds donor (HBD) and acceptors (HBA) (Fig. 6d–f) were similar between the generated molecules and M^pro^ inhibitors. Overall, the similarity between the distributions suggests that the transfer learning process was able to generate molecules in the same physicochemical space as the M^pro^ dataset.

We also investigated the ease of synthesis of the generated molecules. For this analysis, we used the synthetic accessibility score (SAS), which penalizes molecules with highly complex structures, such as high number of stereocenters and multiple ring systems. The SAS score ranges from 1 to 10, with high values being assigned to more complex and difficult to synthesize molecules [49]⁠. The generated molecules had a similar SAS distribution to the training set. The mean SAS of the generated molecules was 2.36, while the training set had a mean of 2.44. Furthermore, the minimum (i.e. lowest complexity) and maximum SAS for the generated set were 1.13 and 6.96, respectively; which were comparable to the minimum (1.12) and maximum (7.81) scores of the training set.

### Navigating the chemical space of the focused library

**Fig. 7:**
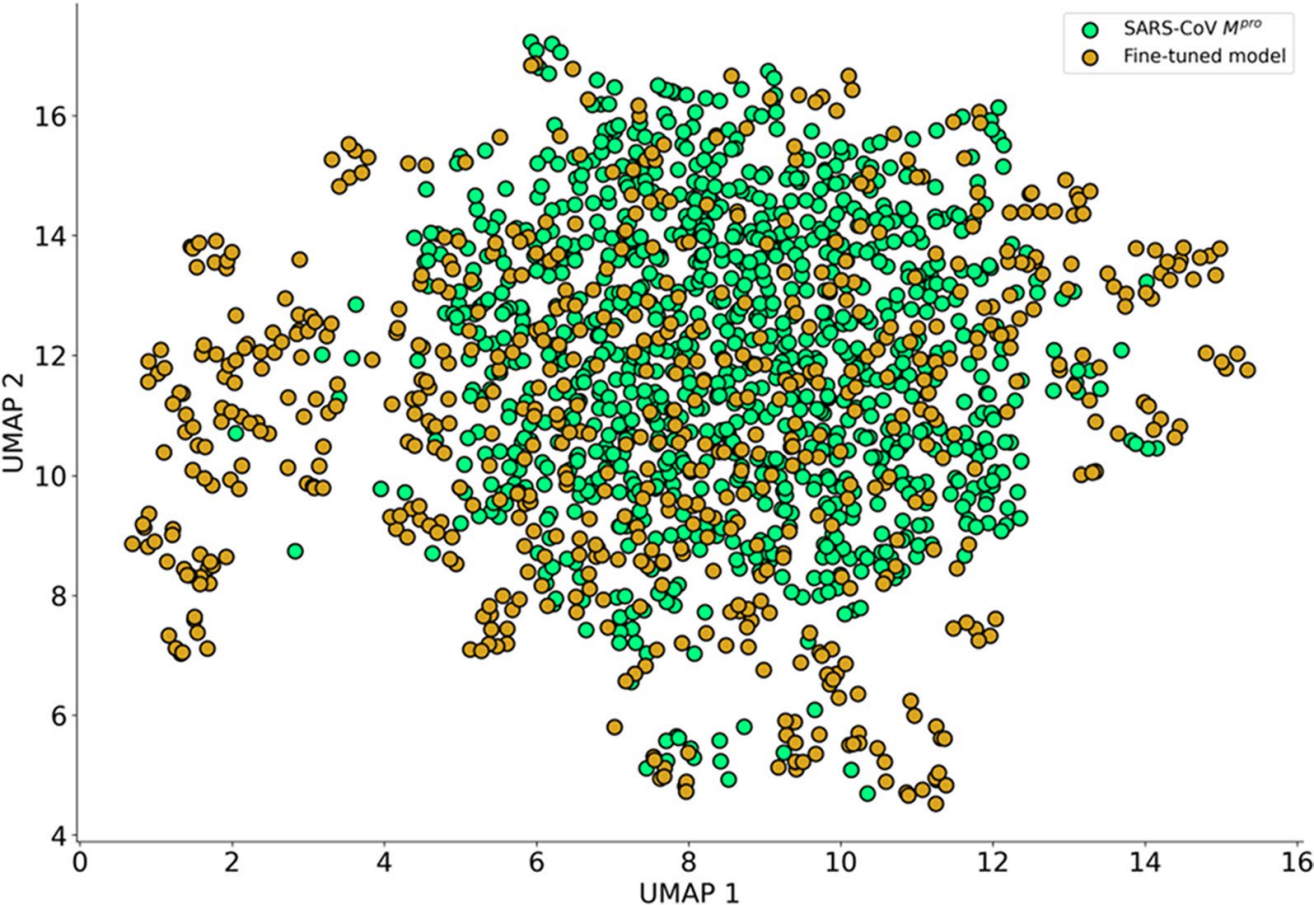
Chemical space of 1000 randomly selected generated molecules (light green) and 629 M^pro^ inhibitors (gold)

To gain a better insight of the fine-tuned chemical space, we calculated the novelty of the 70,000 generated molecules and observed a high proportion (96.46%) of novel molecules compared to M^pro^ inhibitors training set, showing that the transfer learning process did not simply copy molecules from the training data. As shown on the UMAP plots, the generated molecules not only share the chemical space with M^pro^ inhibitors but they extend it by filling gaps with novel molecules, corroborating our previous finding about the similar physicochemical parameters (Fig. 7).

**Fig. 8:**
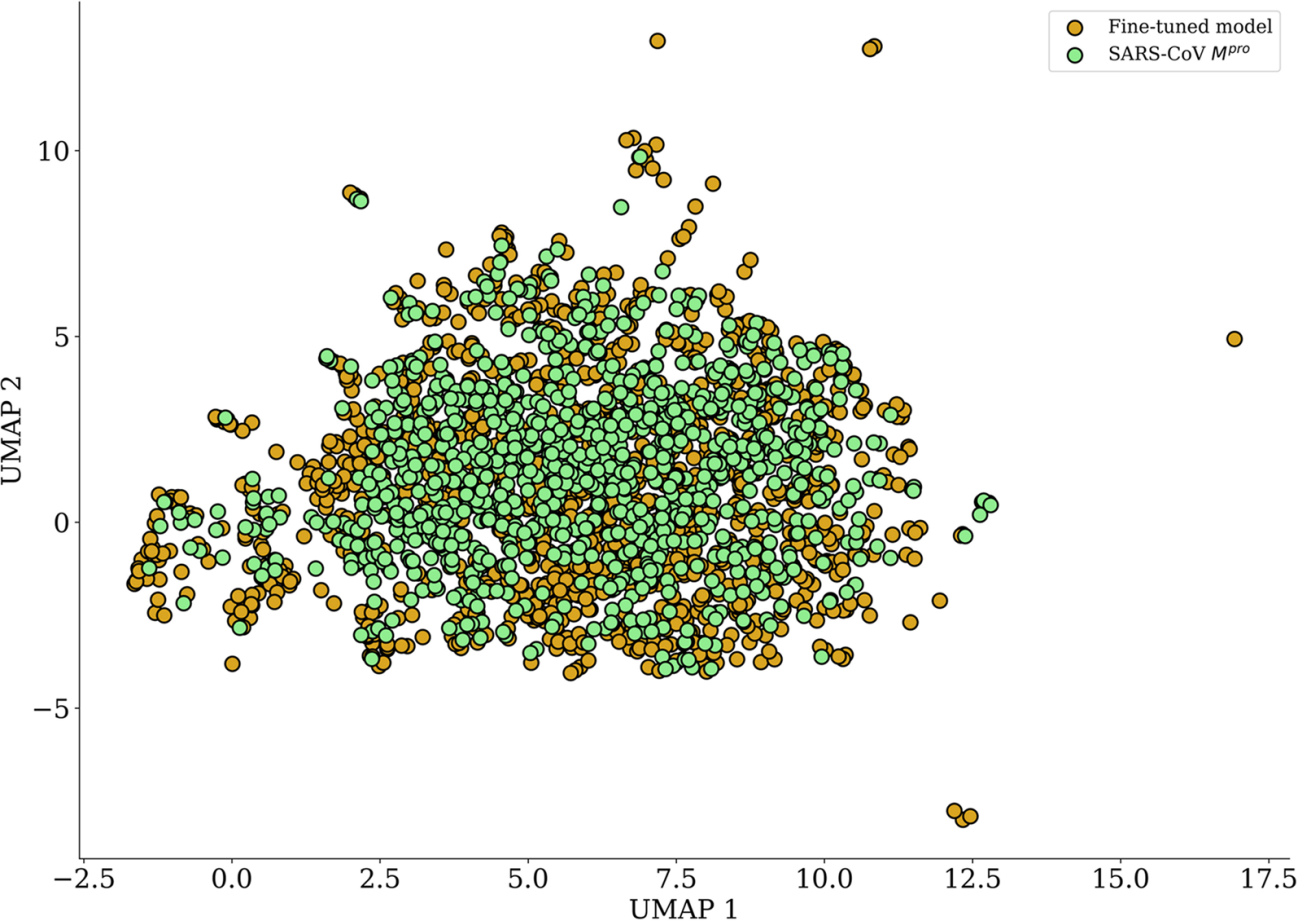
Chemical space of 1000 randomly selected scaffolds from the generated molecules set (light green) and 629 M^pro^ inhibitors (gold)

We also investigated how the generated molecules populated the scaffold (in terms of Bemis-Murcko scaffolds) chemical space, which is an interesting feature for de novo design; if the model could generate novel scaffolds it might be possible to find scaffold hopping opportunities and new chemical series. Among the 70,000 generated molecules, we found 35,713 unique scaffolds (52.89%); of which 35,538 (99.51%) were novel compared to the training set. The UMAP plot shows the overlap in chemical space between Bemis-Murcko scaffolds of the generated molecules and M^pro^ inhibitors. The novel scaffold also filled gaps in chemical space demonstrating that the fine-tuned model successfully approximated the target chemical space (Fig. 8).

**Fig. 9:**
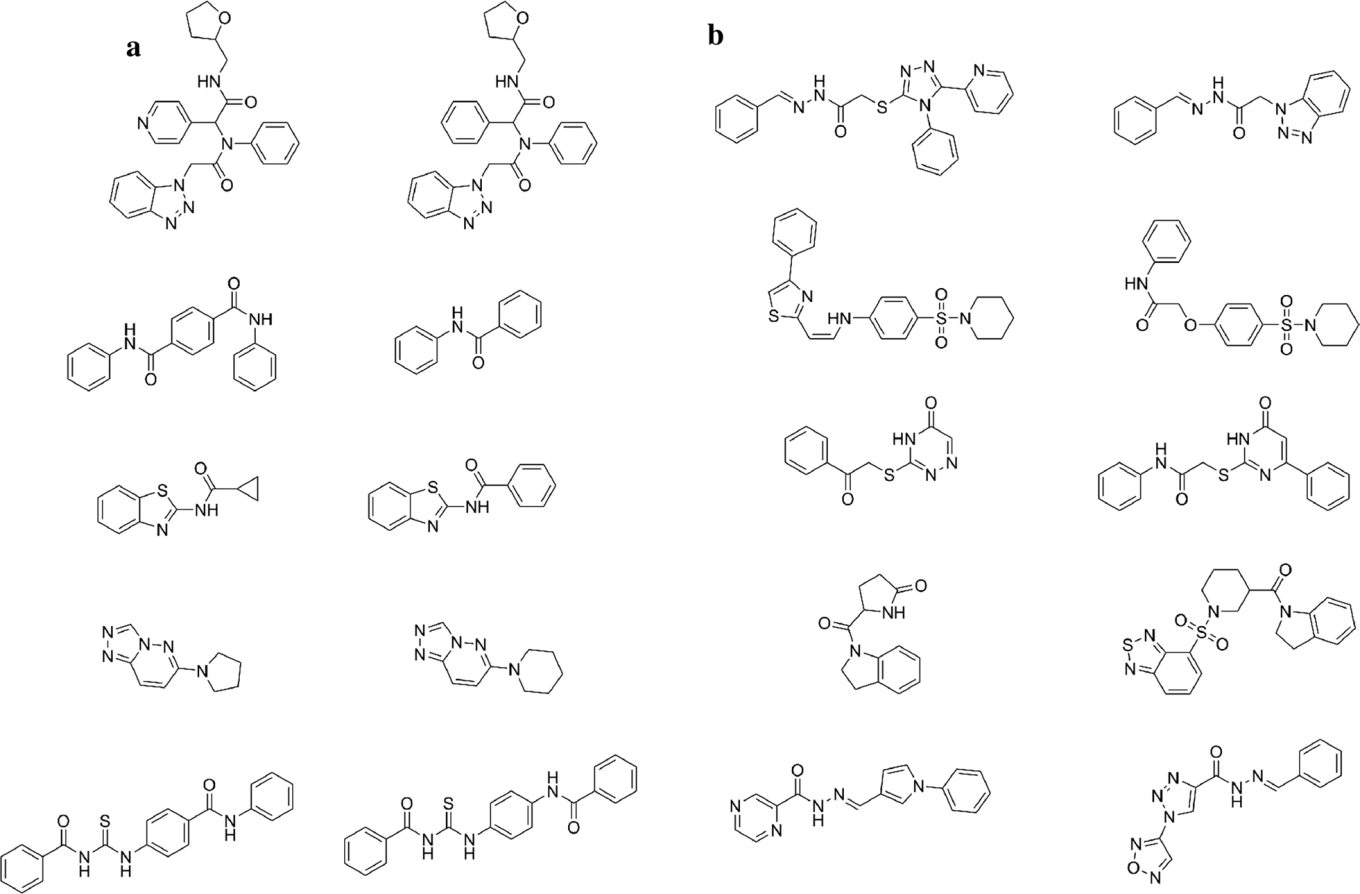
Chemical structures of some generated scaffolds and their closest neighbor on the training set, with small (**a**) and more drastic changes in chemical structure (**b**)

We also analyzed how different the novel scaffolds were from the training set scaffolds. Overall, the novel scaffolds were structurally different to their closest neighbor in the training set, with a Tanimoto coefficient of 0.420 ± 0.10. Some novel scaffolds displayed small modifications compared to their closest neighbors, such as the insertion or removal of a few atoms between rings, the substitution of oxygen for sulfur atoms and substitution of one ring (Fig. 9a). In general, these small modifications did not affect the core of the scaffold, indicating that the model can explore subtle changes while maintaining important features for activity. Some scaffolds showed more drastic modifications, such as replacing atoms of the core of the scaffold, reducing or increasing the complexity of the radicals attached to the core scaffold and changing the core structure completely (Fig. 9b).

In general, the model showed some *creativity*, in a sense that it introduced modifications to existing scaffolds and generated novel structures. This creativity can also be seen in other works. For instance, the RXRs and PPARs inhibitors generated by Merk et al. showed a similar biphenyl scaffold and most modifications were on the radicals. Although the authors did not make the training set available, it is possible that the biphenyl moiety was present on the training set [47]. In another study, Méndez-Lucio and coworkers trained a generative model using chemical (e..g, SMILES strings) and transcriptomic data for 978 genes, showing that the model could generate molecules that were structurally similar to their closest neighbors in the training set, while also introducing a range of modifications to the scaffolds. Concretely, starting with a benzene ring, the authors obtained structures with fused rings, different substitution patterns and also the replacement of carbons atoms to generate heterocycles [50]. A recent approach described by Arús-Pous et al. was used to generate molecules starting from any scaffold; by exhaustively slicing acyclic bonds of the molecules on the training set the authors obtained a vast number of scaffolds and decorators data. After training, the model generated scaffolds decorated with different groups and predicted to be active against dopamine receptor D2 (DRD2). Furthermore, their model could be used to add decorations in a single step or multiple steps [51].

The works summarized above are a small sample of what is possible with modern generative models, showing how different deep learning strategies can be used to generate novel scaffolds with a range of modifications. However, it is important to highlight that the true impact of such modifications in terms of intellectual property and publication quality is still an open question [52].

### Performance of the fine-tuned bioactivity classifier

**Fig. 10:**
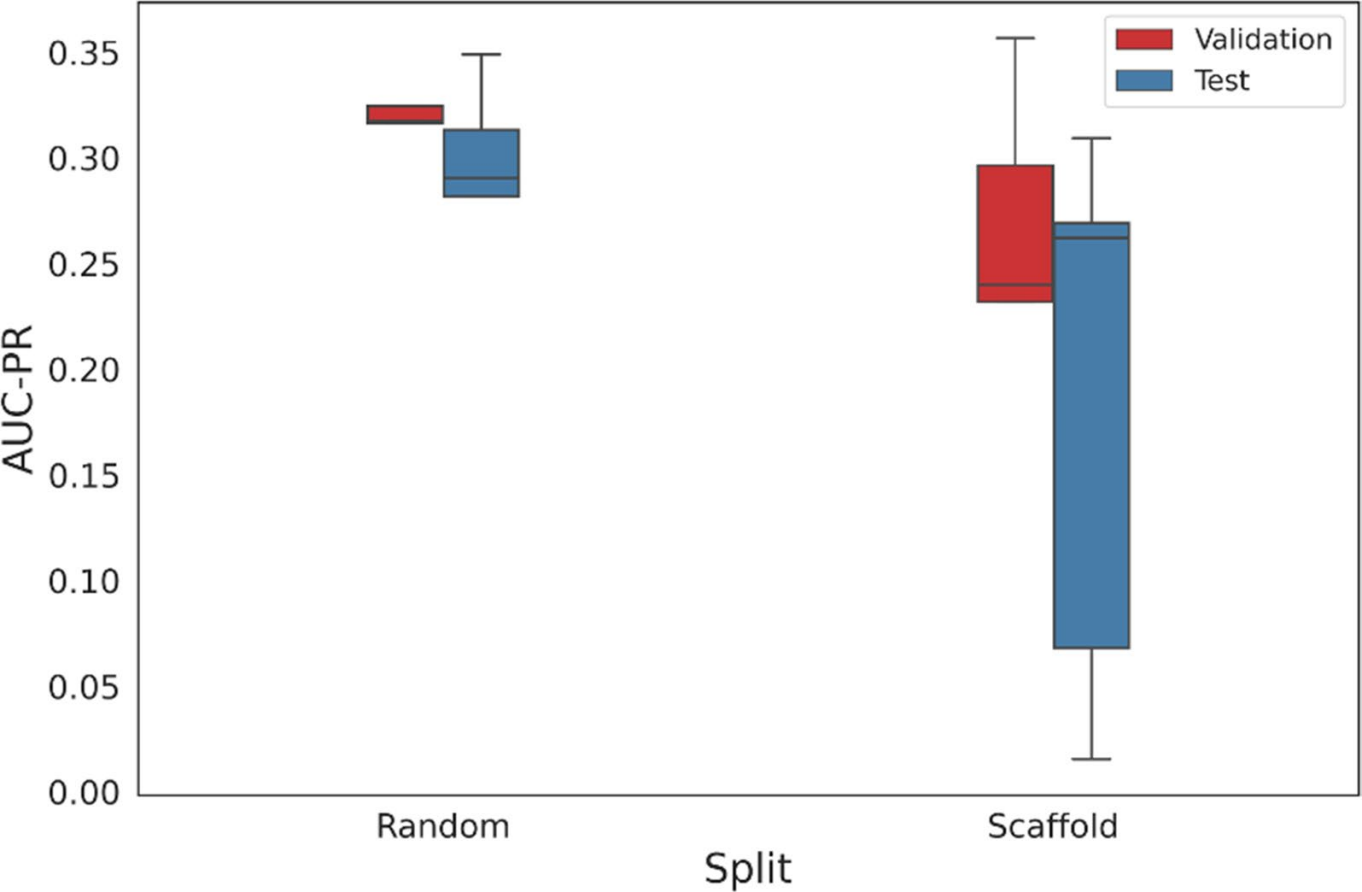
Boxplots of the performance of the classifier grouped by split method. The validation (red bars) and test (blue bars) sets consisted of random or scaffold-based splits of the original SARS-CoV-1 M^pro^ inhibitors data

Having demonstrated that the fine-tuned chemical model approximated the chemical space of M^pro^ inhibitors, we used transfer learning to fine-tune a classification model for bioactivity prediction. The performance of the classifier varied depending on the split method used. For random splitting, the model achieved a validation AUC-PR of 0.310 ± 0.036 and a test AUC-PR of 0.220 ± 0.027. The performance using scaffold split was worse, with validation AUC-PR of 0.251 ± 0.083 and test AUC-PR of 0.185 ± 0.13 (Fig. 10). This drop in performance is expected, since different scaffolds are present in training and validation. However, this method of validation is more realistic when evaluating the performance of the classifier for prospective screening on new scaffolds, since it reduces the bias on specific chemical series [53, 54]. Overall, our model outperformed the baseline AUC-PR for random classification (0.00217), demonstrating it can be used to predict bioactivity for SARS-CoV-1 M^pro^.

**Fig. 11:**
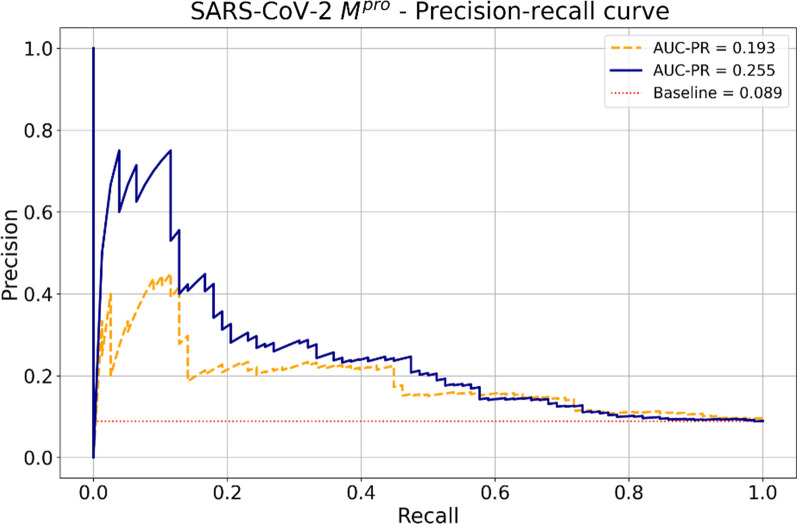
Precision-recall curves for our model (solid blue line) and chemprop (dashed orange line). The baseline area under the precision-recall curve (AUC-PR) for random predictions is given by the ratio of active molecules in the dataset. For the SARS-CoV-2 M^pro^, the baseline was 0.089 and is shown as a dotted red line

We also evaluated the performance of the classifier in predicting the bioactivity of an external set of fragment hits screened against SARS-CoV-2 M^pro^ to estimate its applicability for prospective virtual screening on a similar target. The baseline AUC-PR for randomly predicting hits was 0.089, which is the same as the ratio of hit molecules in the dataset (78 hits × 802 non-hits fragments). Our model clearly outperformed the baseline for random predictions, achieving an AUC-PR of 0.255 (Fig. 11).

We also compared the classifier with the freely available model chemprop, which has been used by the “AI Cures” project to repurpose drugs for SARS-CoV-2 [55]. Chemprop is a message passing neural network (MPNN) that works directly on the molecular graph for molecular property prediction. The predictions are made by averaging the output of 5 models augmented with RDKit features [42]. The precision-recall curve shows that our model outperformed chemprop (Fig. 11).

After analyzing possible thresholds calculated from the precision-recall curve, we decided to use 0.0035 as the probability cutoff to predict a molecule as active to achieve a good balance between precision and recall. Overall, our results suggest that the fine-tuned classifier can be used for prospective virtual screening for SARS-CoV-2 M^pro^.

A limitation of our classifier is that the precision-recall curve shows that it is only possible to achieve a high precision (~ 0.70) at the cost of low recall (< 0.10). In addition, the probabilities output by the classifier were extremely low, with a median of 0.0035. The low probabilities are the result of the extreme class unbalance on the training set, with only 0.1% of active molecules. In future work, we will prioritize calibrating the probabilities to a more reasonable range and improve the recall. The model will be retrained as soon as more activity data is available for SARS-CoV-2 M^pro^ inhibitors. Remarkably, most molecules from AID1706 do not have measured IC_50_ available, since they were classified as active based on the percentage of inhibition on a single concentration screening campaign. Therefore, we still lack confirmatory screening for M^pro^ inhibitors, which would probably improve the performance of deep learning models.

### Predicting the bioactivity of generated molecules

**Fig. 12:**
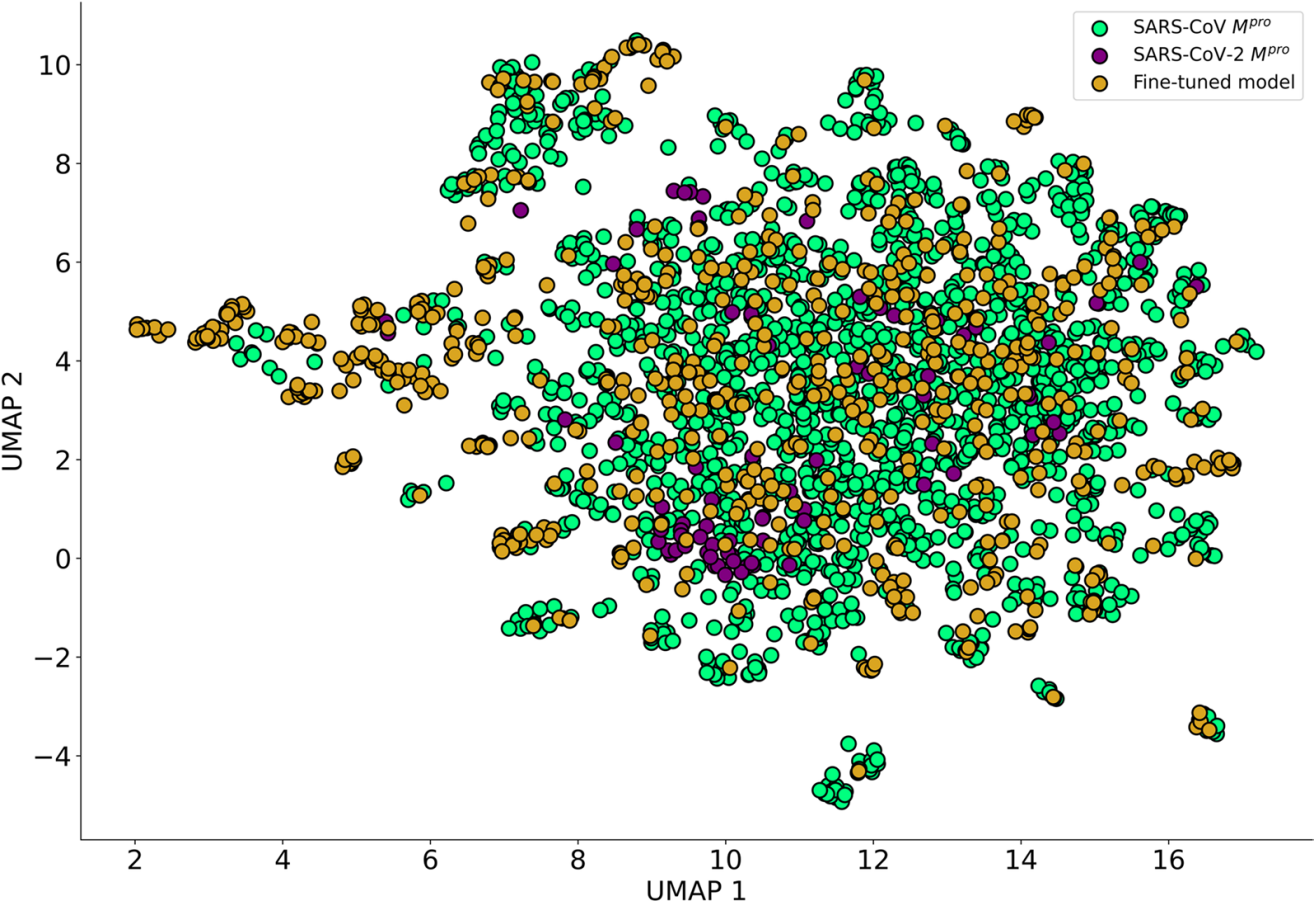
Chemical space of predicted hits (yellow) and M^pro^ inhibitors of SARS-CoV-1 (indigo) and SARS-CoV-2 (light green)

As a proof-of-concept, we used the fine-tuned classifier to predict the bioactivity of the previously generated 70,000 valid SMILES. In total, 1,697 molecules were classified as active and the UMAP plot shows a good overlap between the predicted hits and real M^pro^ inhibitors in chemical space (Fig. 12).

**Fig. 13:**
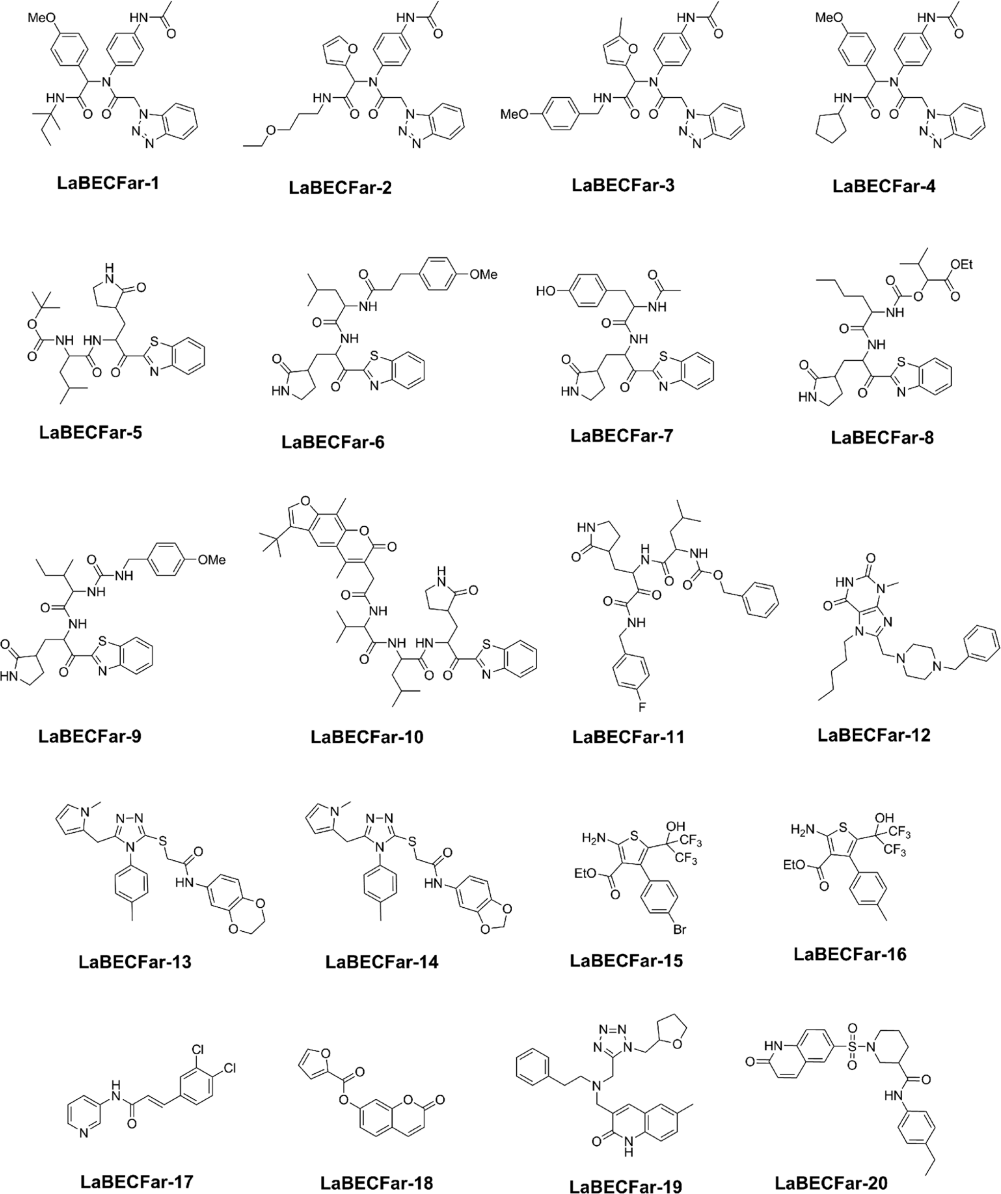
Top 20 predicted active molecules

We also report the top-20 predicted molecules for M^pro^ inhibition (Fig. 13). These 20 molecules were classified as hits with high confidence, with probabilities in the range 0.99–1.0. By analyzing their structures, we found scaffolds that are present in real inhibitors. Of these generated molecules, 5 were rediscovered by our model (**LaBECFar-9, 12, 14, 19** and **20**). Benzotriazoles similar to **LaBECFar-1–4**, have been described as non-covalent inhibitors of SARS-CoV-1 M^pro^ and a X-ray crystal structure between a prototype bound to the enzyme is available on the Protein Databank (PDB: 4MDS) [56]. Peptidomimetic benzothiazolyl ketones, such as **LaBECFar-5–10**, have been described as covalent inhibitors of SARS-CoV-1 M^pro^ [57]. In fact, **LaBECFar-9** is present on the training set and was rediscovered by our approach. The core peptidomimetic structure is preserved in the generated molecules and they also bear the warhead group benzothiazolyl ketone at P1′ position, which could form a covalent bond with the cysteine of the catalytic dyad Cys-His on the active site of M^pro^.

We also applied our classifier to a list of 1959 FDA approved drugs to explore its potential for drug repurposing. Additional file 1: Table S2 shows a pruned list of predicted actives after removing known active molecules that were also present on the training set, including Lopinavir, Boceprevir, Darunavir and Fosamprenavir. These molecules were previously shown to inhibit SAR-CoV-2 M^pro^ in vitro [58–60]. In addition, an X-ray crystal structure of boceprevir complexed with M^pro^ is available on PDB (PDB: 6WNP). Some of the top 10 molecules on Additional file 1: Table S2 have been described in different in silico analysis showing their potential to bind to M^pro^, such as Novobiocin [61], Saquinavir [62, 63], Aprepitant [64] and Leucovorin [65]. Nevertheless, confirmatory screens on these predicted hits are still lacking.

#### Docking simulation

In order to further prioritize molecules for biological testing, we submitted the top-20 predicted hits to a docking simulation using the crystal structure of SARS-CoV-2 M^pro^ (PDB: 6W79). Nine molecules were considered hits, displaying similar binding poses to experimentally validated inhibitors in X-ray crystal complexes with M^pro^. These hits included three benzotriazoles (**LaBECar-1, LaBECFar-3** and **LaBECFar-4**) and four benzothiazolyl ketone (**LaBECFar-5**, **LaBECFar-6**, **LaBECFar-9** and **LaBECFar-7**), one peptidomimetic (**LaBECFar-11**), and one *N*-(2-pyridyl)acetamide derivative (**LaBECFar-17**).

**Fig. 14:**
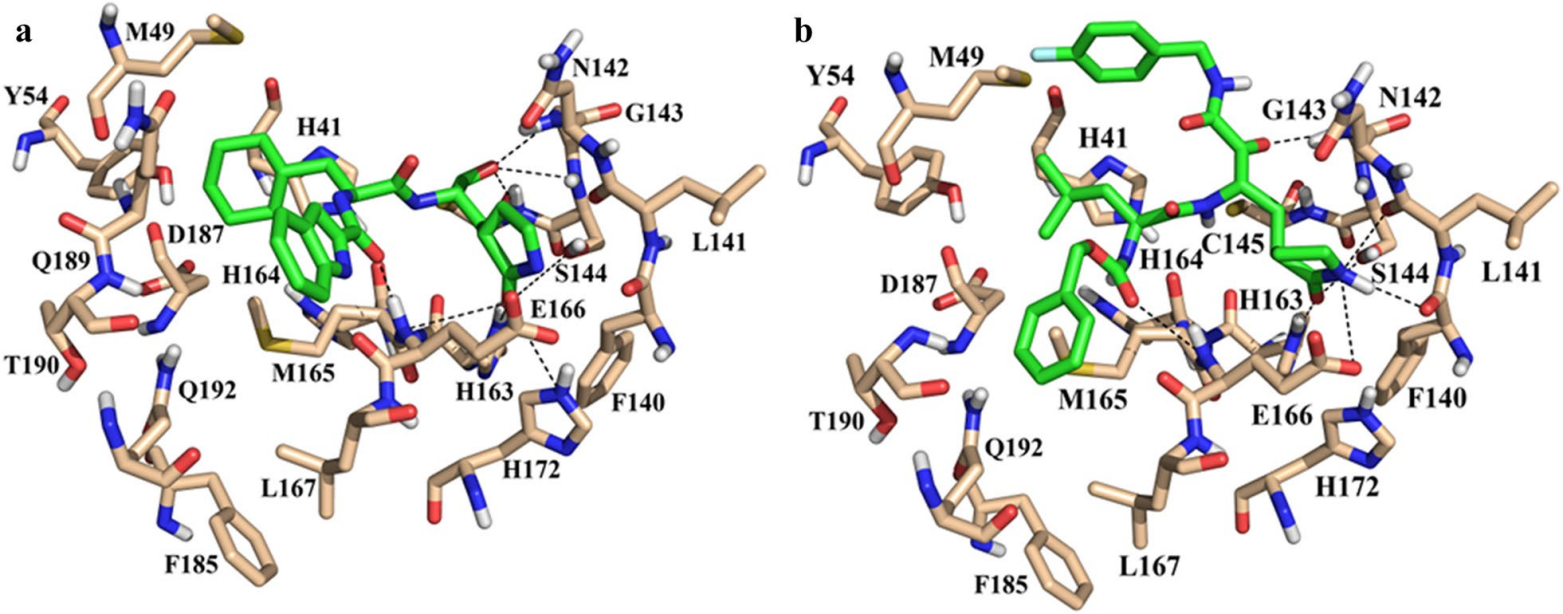
**a** Experimental binding pose of peptidomimetic inhibitor **11a** (PDB: 6LZE) and **b** docked pose of **LaBECFar-11** on SARS-COV-2 M^pro^. (PDB: 6W79). The amino acid residues are shown as beige sticks and the ligands as green sticks

The docked pose of **LaBECFar-11** fits nicely into the active site of M^pro^, showing a similar binding pose to peptidomimetic inhibitors described in other works, including **11a** (Fig. 14a) (PDB: 6LZE) [2, 7, 17, 66, 67]. The γ-lactam group at P1 is a glutamine mimetic and binds in the S1 pocket, with the oxygen atom acting as H-bond acceptor to H163 and the nitrogen as donor to E166 (Fig. 14b). As described in other works, the formation of an H-bond between H163 is critical for activity; H163 is a conserved residue at S1 and is responsible for stabilizing substrates in place via an H-bond with a glutamine residue at position P1 [7, 66]. Interestingly, **LaBECFar-11** does not possess a warhead group at P1′ position, but docking pose suggests it might work as a reversible inhibitor or be optimized for covalent inhibition.

The fluoro-phenylalanine group at P1′ position formed a H-bond with G143 at S1′, adopting a parallel orientation to the S2 pocket. The leucine side chain at position P2 inserted into the S2 pocket and established hydrophobic contacts with the side chains of M49, Y54 and D187 (Fig. 14b). The benzyl carbamate group at P3 position bond on the solvent-exposed S4 pocket, while also forming a H-bond with E166 at S1.

**Fig. 15:**
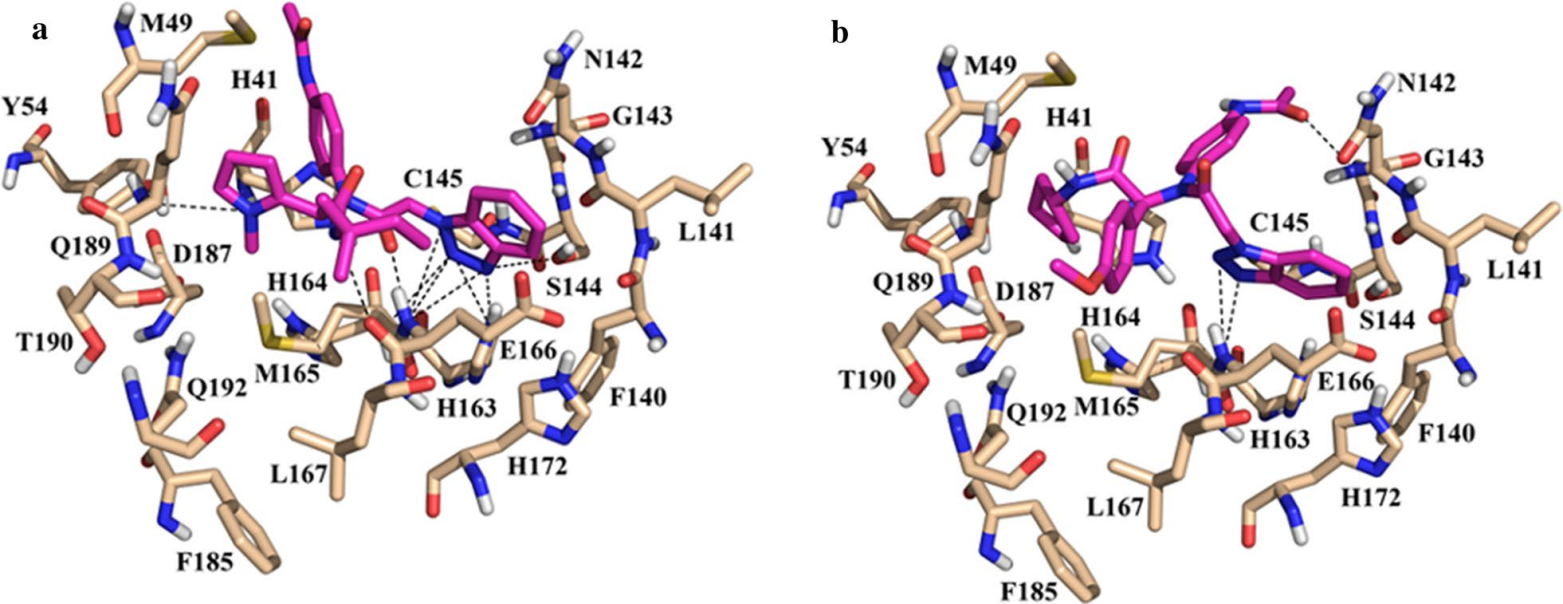
**a** Experimental binding pose of peptidomimetic inhibitor **ML300** (PDB: 4MDS) and **b** docked pose of **LaBECFar-4** on SARS-COV-2 M^pro^. (PDB: 6W79). The amido acid residues are shown as bege sticks and the ligands as pink sticks

The benzotriazole derivatives displayed a similar binding pose to the non-covalent inhibitor **ML300** (PDB: 4MDS) developed by Turlington et al. [68] (Fig. 15a). As shown in Fig. 15b for **LaBECFar-4**, the benzotriazole ring binds to the S1 pocket, formed by the side chains of F140, N142, H163, and H172 (Fig. 15b). Overall, **LaBECar-1** (Additional file 1: Figure S3A), **LaBECFar-3** (Additional file 1: Figure S3B) and **LaBECFar-4** displayed an extensive H-bond network with the S1 pocket, with H163 and E166 representing the main residues stabilizing the ligand.

The S2 pocket also hosted a series of interactions with the **LaBECar-1**, **LaBECFar-3** and **LaBECFar-4**. The cyclopentyl moiety of **LaBECar-4** inserted into S2 and stacked with the imidazole ring of H41 (Fig. 15b). The cyclopentyl moiety also made extensive hydrophobic contacts with M49, Y54 and D187 at S2. The same interaction pattern of hydrophobic interactions was observed for LaBECFar-1 (Additional file 1: Figure S3A) and 3 (Additional file 1: Figure S3B). The *N*-(2-phenyl)-acetamide group at position P1 in **LaBECar-1** (Additional file 1: Figure S3A) and 3 (Additional file 1: Figure S3B) was solvent exposed, protruding from the binding site without any noticeable interactions with M^pro^. The same orientation was not observed in **LaBECFar-4**, where the *N*-(2-phenyl)-acetamide group was accommodated between the S2/S1′ pockets and established an H-bond with G143 (Fig. 15b), which is similar to the pose of inhibitor **ML300** [68].

Different groups were positioned on the solvent-exposed S4, which is in accordance with the high tolerance of this subsite to a range of functional groups [7, 17, 68]. It might be possible to truncate **LaBECar-1**, **LaBECFar-3** and **LaBECFar-4** and reduce the molecular weight by removing the P3 group at S4, since it is exposed to solvent. A similar strategy was adopted by Turlington et al., for the development and optimization of **ML300** and other benzotriazole derivatives [68].

**Fig. 16:**
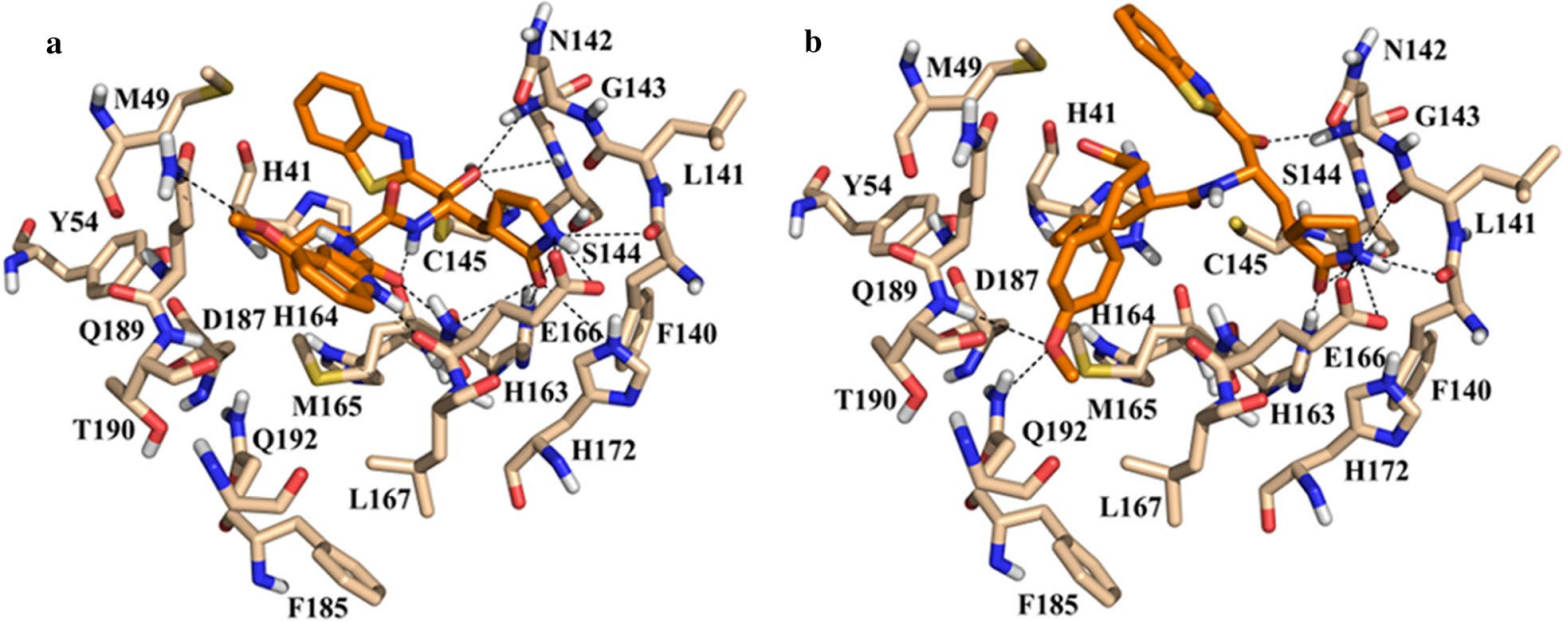
**a** Experimental binding pose of peptidomimetic inhibitor **GRL-0240-20** (PDB: 6XR3) and **b** docked pose of **LaBECFar-5** on SARS-COV-2 M^pro^. (PDB: 6W79). The amido acid residues are shown as beige sticks and the ligands as orange sticks

The benzothiazolyl ketones **LaBECFar-5** (Fig. 16b), **LaBECFar-6** (Additional file 1: Figure S4A), **LaBECFar-7** (Additional file 1: Figure S4B), **LaBECFar-9** (Additional file 1: Figure S4C) displayed a binding pose that could favour covalent inhibition, with the carbonyl positioned 4.2 Å from the sulfur atom of C145 at S1′. As shown in Fig. 16b for **LaBECFar-5**, the binding pose is similar to the recently solved X-ray crystal complex between the benzothiazolyl inhibitor **GRL-0240-20** (Fig. 16a) and SARS-CoV-2 M^pro^ (PDB: 6XR3). The γ-lactam group at P1 position established H-bond with the imidazole of H163 and the side chain of E166 on the S1 subsite. The leucine side chain at P2 inserted into the S2 pocket and formed hydrophobic interactions with M49, D187 and Y54. The 4-methoxy-benzyl group at P3 interacted with the solvent-exposed S4, forming a H-bond with Q192.

**Fig. 17:**
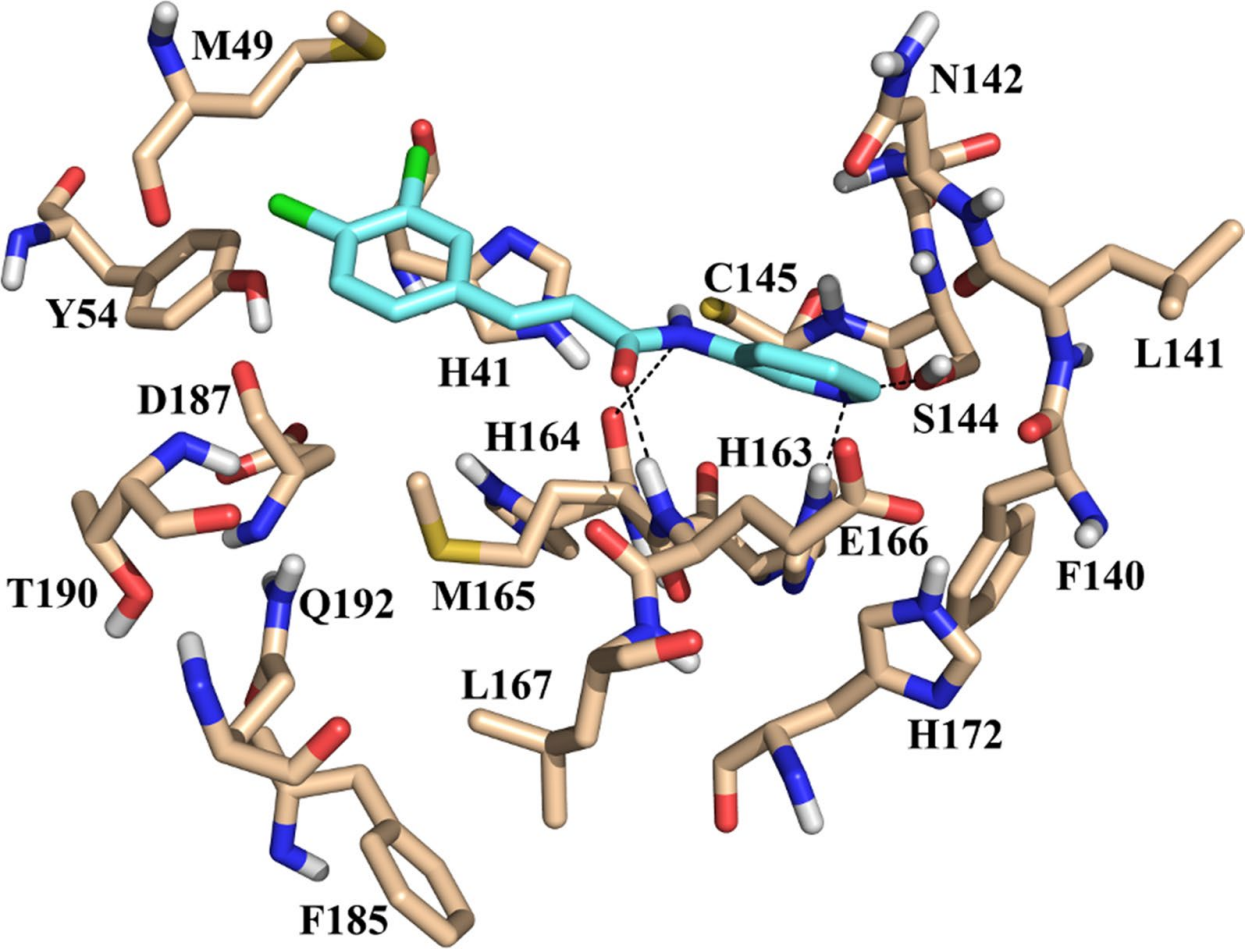
Docked pose of **LaBECFar-17** on SARS-COV-2 M^pro^. (PDB: 6W79). The amido acid residues are shown as beige sticks and the ligand is shown as light blue sticks

We also report the **LaBECFar-17**; bearing an acrylamide moiety that could covalently inhibit M^pro^ (Fig. 17). In fact, one acrylamide from the training set (Pubchem SID: 47,196,538) is an analogue of **LaBECFar-17** and was confirmed to be active on two Pubchem confirmatory screenings against SARS-CoV-1 M^pro^ (AIDs: 1879 and 1944). The docked pose of **LaBECFar-17** revealed that the 3,4-dichloro group inserts into the S2 pocket, while the pyridine ring forms a H-bond with H163 at S1 (Fig. 17). The warhead acrylamide is at 5.9 Å from the catalytic C145 and forms H-bonds with E166 and H164 at S1.

#### PAINS filtering

In a final round of in silico analysis, we submitted the top-20 predicted hits to a Pan Assay Interference Compounds (PAINS) filter implemented in the FAF-Drugs4 server in order to identify molecules with the potential to interfere with biological assays. Not surprisingly, LaBECFar-15 and 16 were flagged as PAINS; the amino-thiophene group in these molecules is known to have thiol reactivity. The other 18 predicted hits passed in all PAINS filters.

In silico filtering using predefined rules is a valuable tool to prioritize molecules from huge databases and reduce the risks of false positives in biological assays [69, 70]. Many tools are available for free and implemented in packages such as RDKit and servers such as FAF-Drugs4. However, automatic virtual filters are not magic bullets to catch all molecules that could interfere with assays [71]. For instance, current strategies to develop M^pro^ inhibitors often rely on warhead groups, such as α,β-unsaturated carbonyls, aldehydes and thiol-reactive esters [72, 73]; these groups would probably be flagged as problematic in some PAINS filters [70, 74, 75]. Therefore, the selection of molecules for follow up analysis should be done carefully and must take into account the nature of the target and the known inhibitors available. These highly reactive molecules could be problematic to optimize, but they are still the most abundant source of starting points to develop M^pro^ inhibitors and should not be discarded before experimental confirmation of bioactivity and that they are not interfering with the biological assay.

## Conclusions

We used ULMFit to train a chemical model for de novo design and fine-tune a classifier for bioactivity prediction on SARS-CoV-2 M^pro^. The chemical space of the generated molecules overlapped with the target chemical space of M^pro^ inhibitors, showing that the key structural features were properly captured by the model. In addition, the generated molecules and real M^pro^ inhibitors showed similar physicochemical properties.

The fine-tuned classifier outperformed the random classification baseline and a model that is being used to repurpose drugs for SARS-CoV-2 M^pro^. The predicted active molecules also shared scaffolds with real M^pro^ inhibitors, while introducing a range of changes to lateral chains and the core of the scaffolds, indicating it could be used to explore the structure–activity of chemical series.

We also highlight that the current version of our generative model is still limited by the nature of the training data. As more molecules are screened against M^pro^, we will update the model in order to generate more diverse and novel molecules.

## Supplementary Information

**Additional file 1:** **Table S1**. Validity, uniqueness and novelty (mean ± std) of SMILES generated after training. We sampled 10,000 SMILES for eachtemperature (2,000 SMILES in five independent runs). **Figure S1**. UMAP plot of the chemical space of scaffolds generated by the general chemical modeland scaffolds from ChEMBL (2,000 molecules were randomly selected for each set). **Figure S2**. Redocking experiment to validate the molecular dockingprotocol. The docked pose of ligand X77 from SARS-COV-2 M^pro^ (PDB: 6W79) is shown as purple sticks and the experimental binding pose as greensticks. The enzyme surface is shown in bege. The RMSD between the docked and experimental pose was 1.106 Å. **Figure S3**. Docked poses of LaBECFar-1and LaBECFar-3 on SARS-COV-2 M^pro^. (PDB: 4MDS). The amino acid residues are shown as bege sticks and the ligands are shown as pink sticks.**Figure S4**. Docked poses of LaBECFar-6, LaBECFar-7 and LaBECFar-9 on SARS-COV-2 M^pro^. (PDB: 6W79). The amido acid residues are shown asbege sticks and the ligands are shown as orange sticks. **Table S2**. FDA approved drugs predicted to be active on SARS-CoV-2 M^pro^.

## Data Availability

The datasets, cross validation splits and a template Jupyter notebook to train the models during the current study are available in the Github repository, https://github.com/marcossantanaioc/De_novo_design_SARSCOV2.

## Abbreviations

ULMFit: Universal language model fine-tuning
M^pro^: Main protease
SARS-CoV-2: Severe acute respiratory syndrome coronavirus 2
COVID-19: Coronavirus disease 2019
NLP: Natural language processing
LSTM: Long short-term memory
RNN: Recurrent neural network
AWD-LSTM: ASGD weight-dropped LSTM
SMILES: Simplified molecular input line entry specification
BOS: Beginning of string
EOS: End of string
GPU: Graphical processing unit
SAR: Structure–activity relationship
QSAR: Quantitative structure–activity relationship
SAS: Synthetic accessibility score
HBA: Hydrogen bond acceptor
HBD: Hydrogen bond donor
MW: Molecular weight
ECFP: Extended connectivity fingerprint
MACCS: Molecular access system
Se: Sensibility or recall
Sp: Specificity
Pre: Precision
IC_50_: Half maximal inhibitory concentration
UMAP: Uniform manifold approximation and projection
PAINS: Pan assay interference compounds
NSP: Nonstructural protein
ORF: Open reading frame
VAE: Variational autoencoders
GAN: Generative adversarial networks
AAE: AAdversarial Autoencoders
PPARγ: Peroxisome proliferator-activated receptor gamma
AUC-PR: Area under the precision-recall curve
MPNN: Message passing neural network
