# Supplementary material for: De novo design and bioactivity prediction of SARS-CoV-2 main protease inhibitors using recurrent neural network-based transfer learning
Source: BMC Chem. 2021 Feb 2;15(1):8. doi: 10.1186/s13065-021-00737-2 (PMC7852053; doi:10.1186/s13065-021-00737-2)
Supplement: Supplementary file 1 — Additional file 1: Table S1. Validity, uniqueness and novelty (mean ± std) of SMILES generated after training. We sampled 10,000 SMILES for eachtemperature (2,000 SMILES in five independent runs). Figure S1. UMAP plot of the chemical space of scaffolds generated by the general chemical modeland scaffolds from ChEMBL (2,000 molecules were randomly selected for each set). Figure S2. Redocking experiment to validate the molecular dockingprotocol. The docked pose of ligand X77 from SARS-COV-2 Mpro (PDB: 6W79) is shown as purple sticks and the experimental binding pose as greensticks. The enzyme surface is shown in bege. The RMSD between the docked and experimental pose was 1.106 Å. Figure S3. Docked poses of LaBECFar-1and LaBECFar-3 on SARS-COV-2 Mpro. (PDB: 4MDS). The amino acid residues are shown as bege sticks and the ligands are shown as pink sticks.Figure S4. Docked poses of LaBECFar-6, LaBECFar-7 and LaBECFar-9 on SARS-COV-2 Mpro. (PDB: 6W79). The amido acid residues are shown asbege sticks and the ligands are shown as orange sticks. Table S2. FDA approved drugs predicted to be active on SARS-CoV-2 Mpro. [file 13065_2021_737_MOESM1_ESM.docx]

**Additional file 1**

***De novo Design and Bioactivity Prediction of SARS-CoV-2 Main Protease Inhibitors using Recurrent Neural Network-based Transfer Learning***

***Marcos V. S. Santana ^1[0000-0003-0204-9396]^*** ***, Floriano Paes Silva-Junior ^1,* [0000-0003-4560-1291]^***

^1^ LaBECFar – Laboratório de Bioquímica Experimental e Computacional de Fármacos, Instituto Oswaldo Cruz, Fundação Oswaldo Cruz, Rio de Janeiro, Brazil.

* Corresponding author: floriano@ioc.fiocruz.br. LaBECFar – Laboratório de Bioquímica Experimental e Computacional de Fármacos, Instituto Oswaldo Cruz, Fundação Oswaldo Cruz, Rio de Janeiro, RJ 21040-900, Brazil

**Part I - Universal Language Model Fine-tuning**

1. **THEORY**

Instead of searching chemical databases for potential antivirals, in this work we propose a deep learning platform to generate new chemical matter focused on SARS-CoV-2 Mpro. Our method is based on the ULMFit (Universal Language Model Fine-Tuning) approach developed by Howard and Ruder to address the problem of transfer learning in natural language processing (NLP) classification tasks [[1]](https://paperpile.com/c/gDxyQ1/6YgY). Specifically, ULMFit allows pre-training a general-domain model and then fine-tuning it on a target task. The approach can be divided into three parts:

1. Initially, a general-domain model is trained on a large text corpus to learn to predict the next word (or character) in a sentence. Since this task is strongly dependent on the model learning long and short-term dependencies between the words, we can think of training the model to learn a very general idea of how a language works. In a chemical sense, this translates to learning how to build valid SMILES strings of molecules.
2. The features learned by the pre-trained model can be fine-tuned to adapt to the idiosyncrasies of a target task.
3. In the last step, the fine-tuned model can be used as part of a classification model to predict the bioactivity on Mpro.

The pre-training and fine-tuning of chemistry-based language models, or generative models, is a growing research field for *de novo drug* design [[2, 3]](https://paperpile.com/c/gDxyQ1/M8bLS+LHCC7). The main idea of this kind of deep learning model is to use a neural network that can generate new chemical matter after seeing examples of valid molecules. One of the most used architectures for this are recurrent neural networks (RNN). RNN’s are neural networks that can deal with sequences of variable length, such as the ones in natural language processing [[4]](https://paperpile.com/c/gDxyQ1/g2Xq6), audio [[5]](https://paperpile.com/c/gDxyQ1/04UVI) and video [[6]](https://paperpile.com/c/gDxyQ1/xNLNI) tasks. The recurrent operation is the heart of RNN; each item in a sequence serves as input to the neural net in order to predict the next item in the sequence [[7, 8]](https://paperpile.com/c/gDxyQ1/G5Irh+EvuxN). RNN’s can also learn long and short-term dependencies between items and learn how the sequence is structured [[9]](https://paperpile.com/c/gDxyQ1/SM3o1).

RNN’s are suitable to work with molecules because most molecular information is available as text files; in SMILES strings each character contains some information about the molecule and RNN’s could learn the *chemical language* by looking at a number of examples [[2]](https://paperpile.com/c/gDxyQ1/M8bLS). Concretely, different studies have demonstrated that RNN’s can be trained on SMILES strings of a large molecule collection to generate molecules that are similar to active molecules of a target chemical space [[7, 8, 10–13]](https://paperpile.com/c/gDxyQ1/xHjOG+G5Irh+clyzQ+F7Fgl+EvuxN+dFs9X). In addition, these models can be combined with reinforcement learning (RL) to optimize them towards physico-chemical and biological properties of interest [[14–19]](https://paperpile.com/c/gDxyQ1/MSrOc+HGe0c+eHoF8+BEMRc+hCcLF+8mMLu).

In this work we used ULMFiT [[1]](https://paperpile.com/c/gDxyQ1/6YgY) to train a chemistry model to generate molecules in the same chemical space as molecules screened against SARS-CoV main protease (Mpro); and a classification model to predict the bioactivity of the generated molecules on SARS-CoV-2 Mpro. The molecules predicted as active were further analysed using molecular docking to investigate possible interactions with Mpro.

**Part II - General Chemical Model Validation**

**1. General Chemical Model Validation.** We initially validated the chemical model trained on ChEMBL to access its potential to generate molecules using SMILES strings (Table 1). The main metrics have been used to validate generative models in other works [31,34,55](https://paperpile.com/c/zFZPEu/6kBxF+H3XwD+Gksjf).

**Table S1.** Validity, uniqueness and novelty (mean ± std) of SMILES generated after training.

| **Temperature*** | **Validity (%)** | **Uniqueness (%)** | **Novelty (%)** |
| --- | --- | --- | --- |
| 0.20 | 99.27 ± 0.21 | 35.26 ± 1.56 | 83.01 ± 1.06 |
| 0.50 | 99.81 ± 0.050 | 95.78 ± 0.37 | 77.73 ± 0.31 |
| 0.60 | 99.74 ± 0.11 | 98.70 ± 0.37 | 80.43 ± 0.31 |
| 0.70 | 99.30 ± 0.18 | 99.09 ± 0.23 | 83.11 ± 0.42 |
| 0.75 | 98.96 ± 0.31 | 98.81 ± 0.34 | 84.25 ± 1.20 |
| 0.80 | 98.73 ± 0.15 | 98.69 ± 0.17 | 86.57 ± 0.36 |
| 1.00 | 94.26 ± 0.48 | 94.24 ± 0.46 | 92.14 ± 0.59 |
| 1.20 | 81.80 ± 1.23 | 81.78 ± 1.20 | 95.72 ± 0.60 |

*We sampled 10,000 SMILES for each temperature (2,000 SMILES in five independent runs).

As expected, the proportion of valid SMILES decreased steadily with temperature when T ≥ 0.75. As the randomness of sampling increases, the number of valid molecules decreases; which is consistent with previous works [33,34](https://paperpile.com/c/zFZPEu/9kgh4+Gksjf) (Table S1). Most errors were associated with incomplete ring systems, where RDKit could not find matching pairs of brackets on the SMILES string, and a smaller proportion consisted of invalid valances, such as C^+5^ and Cl^+2^.

When *T* = 0.20, the generated SMILES were mostly long acyclic molecules with few branches and enriched with carbon and carbonyl / amide groups, showing that the model is making high confidence predictions about the next atom based on the previous tokens. This is not surprising since carbon, oxygen and nitrogen are the most prevalent tokens on the training data.

Increasing the temperature resulted in higher uniqueness (or diversity), with the maximum value achieved with *T* = 0.70 (uniqueness = 99.09 ± 0.23%). When *T* > 0.70, there was a progressive decrease in diversity, with *T* = 1.2 returning the lowest score (81.78 ± 1.20%). This lower diversity could also be a reflection of the lower proportion of valid SMILES in higher temperatures. Despite the drop in diversity, all temperatures still yielded more than 70% unique SMILES. The novelty score also increased with temperature, achieving the highest value with *T* = 1.2, indicating the model is not simply copying molecules from the training set but in fact generating new chemical matter.

For all temperatures, a few very complex molecules were generated, such as 9-members rings and polycyclic compounds, which is probably a reflection of the general nature of the model since it was trained on ~1.6 million molecules from ChEMBL and we did not impose restrictions to molecular complexity, except the maximum size of the SMILES string to generate (i.e., 140 characters).

Overall, our results indicate that the model can generate diverse and novel molecules. As shown in Table 1, a good compromise of validity, diversity and novelty was obtained when sampling with *T* = 0.8. Therefore, we decided to use *T* = 0.8 for the subsequent experiments.

***1.1. Scaffold Chemical Space***


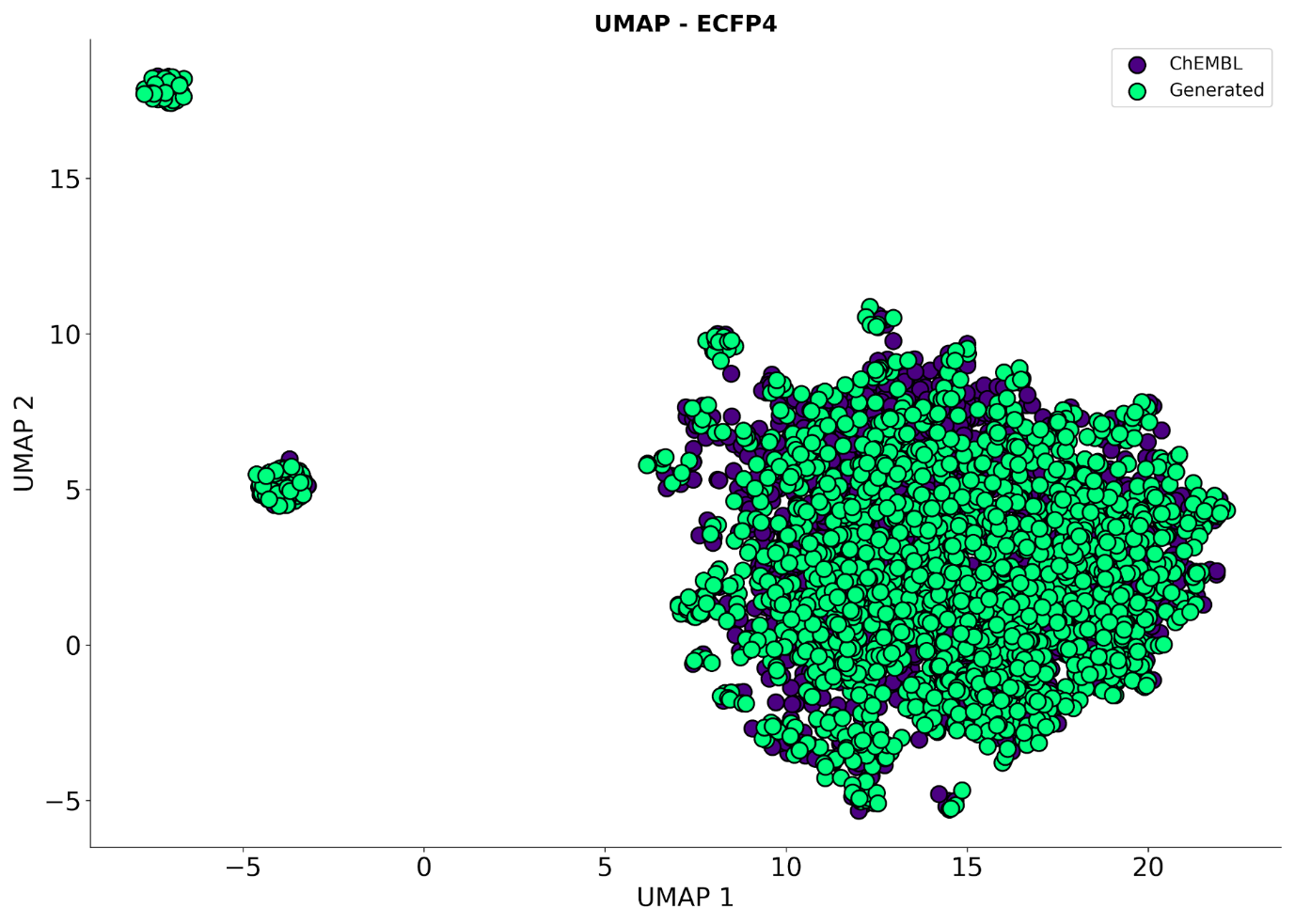


**Figure S1.** UMAP plot of the chemical space of scaffolds generated by the general chemical model and scaffolds from ChEMBL (2,000 molecules were randomly selected for each set).

**Part III - Molecular docking**

**
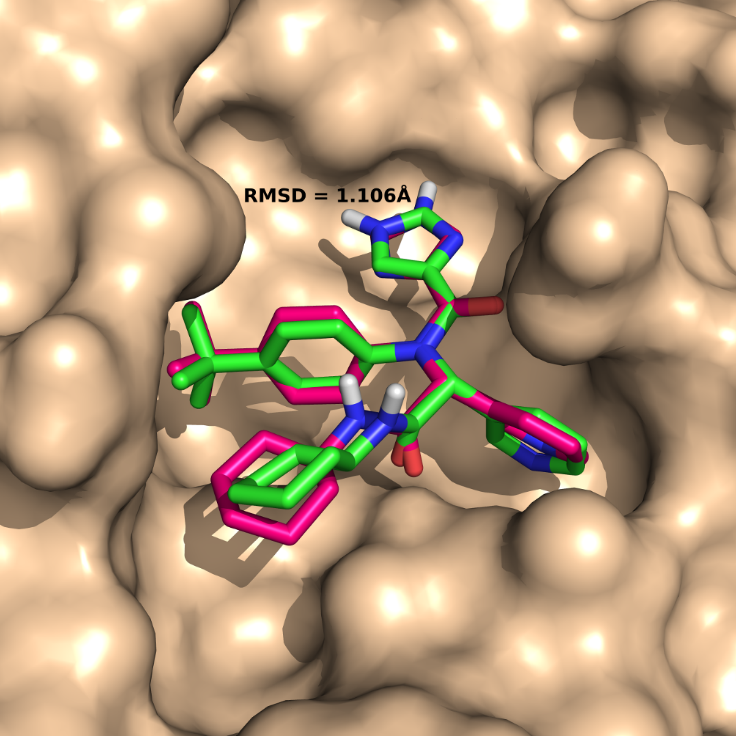
**

**Figure S2**. Redocking experiment to validate the molecular docking protocol. The docked pose of ligand **X77** from SARS-COV-2 M^pro^ (PDB: 6W79) is shown as purple sticks and the experimental binding pose as green sticks. The enzyme surface is shown in bege. The RMSD between the docked and experimental pose was 1.106Å.

**
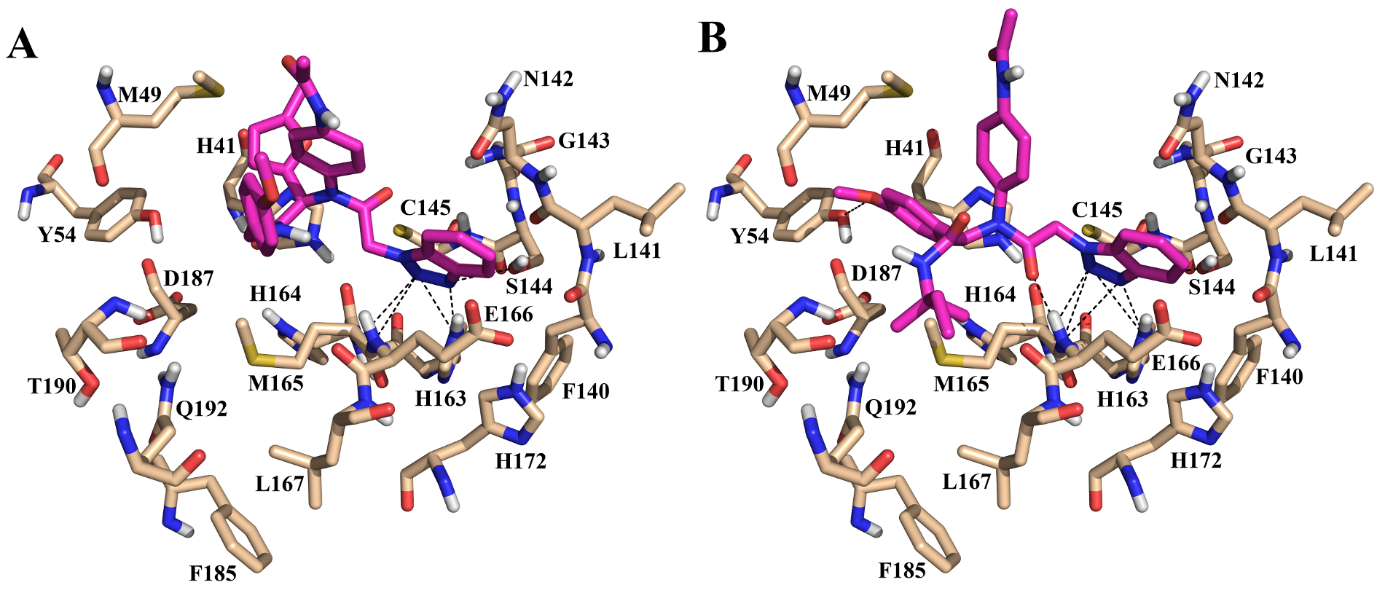
**

**Figure S3**. Docked poses of **LaBECFar-1** and **LaBECFar-3** on SARS-COV-2 M^pro^. (PDB: 6W79). The amido acid residues are shown as bege sticks and the ligands are shown as pink sticks.

**
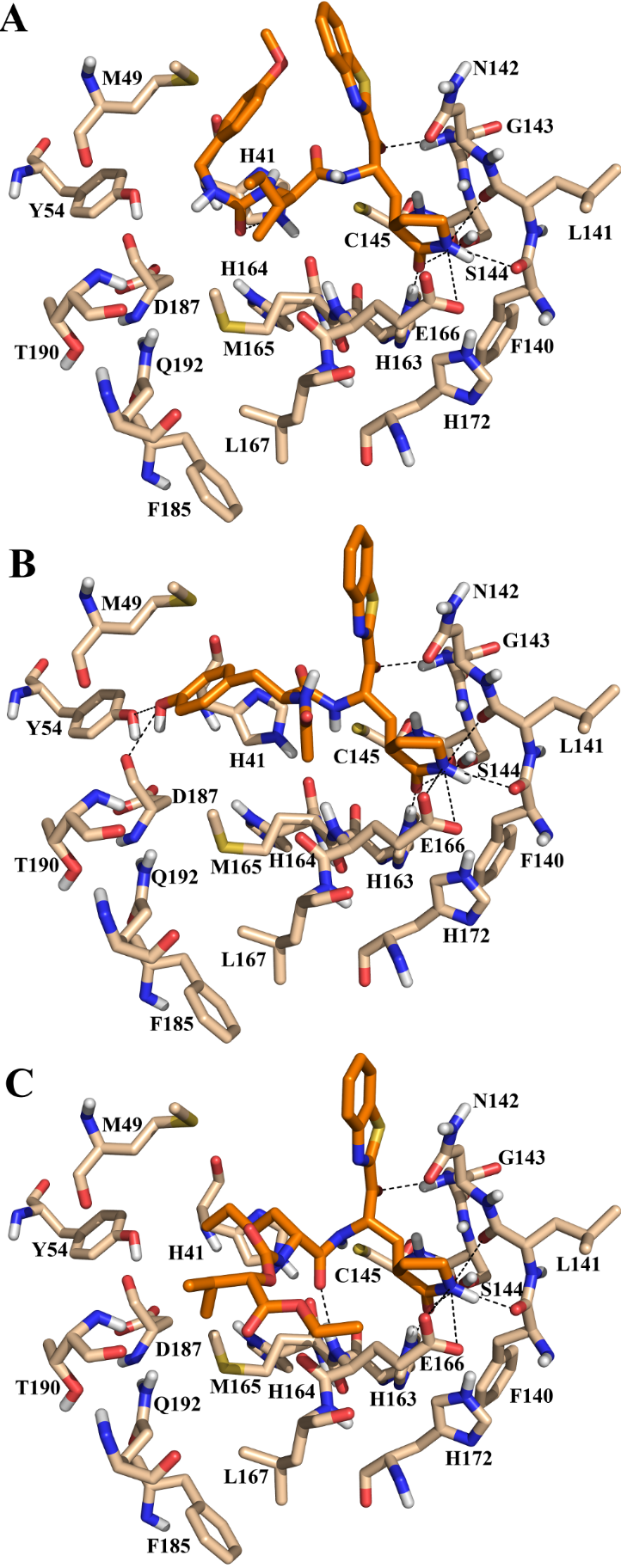
**

**Figure S4**. Docked poses of **LaBECFar-6,** **LaBECFar-7** and **LaBECFar-9** on SARS-COV-2 M^pro^. (PDB: 6W79). The amido acid residues are shown as bege sticks and the ligands are shown as orange sticks.

**Part IV - Drug Repurposing**

1. **Exploring the potential of the M^pro^ classifier for drug repurposing**

Table S2. FDA approved drugs predicted to be active on SARS-CoV-2 M^pro^.

| **Name** | **Smiles** | **Probability** |
| --- | --- | --- |
| Novobiocin | CO[C@@H]1[C@@H](OC(N)=O)[C@@H](O)[C@H](Oc2ccc3c(O)c(NC(=O)c4ccc(O)c(CC=C(C)C)c4)c(=O)oc3c2C)OC1(C)C | 1.000 |
| Saquinavir | CC(C)(C)N=C(O)[C@@H]1C[C@@H]2CCCC[C@@H]2CN1C[C@@H](O)[C@H](Cc1ccccc1)N=C(O)[C@H](CC(=N)O)NC(=O)c1ccc2ccccc2n1 | 0.998 |
| Ioxaglic acid | CNC(=O)c1c(I)c(C(=O)NCC(=O)Nc2c(I)c(C(=O)O)c(I)c(C(=O)NCCO)c2I)c(I)c(N(C)C(C)=O)c1I | 0.885 |
| Gemifloxacin | CO/N=C1\CN(c2nc3c(cc2F)c(=O)c(C(=O)O)cn3C2CC2)CC1CN | 0.867 |
| Tipranavir | CCC[C@@]1(CCc2ccccc2)CC(O)=C([C@H](CC)c2cccc(NS(=O)(=O)c3ccc(C(F)(F)F)cn3)c2)C(=O)O1 | 0.716 |
| Remikiren | CC(C)(C)S(=O)(=O)C[C@@H](Cc1ccccc1)C(O)=N[C@@H](Cc1cnc[nH]1)C(O)=N[C@@H](CC1CCCCC1)[C@@H](O)[C@@H](O)C1CC1 | 0.395 |
| Aprepitant | C[C@@H](O[C@H]1OCCN(Cc2n[nH]c(=O)[nH]2)[C@H]1c1ccc(F)cc1)c1cc(C(F)(F)F)cc(C(F)(F)F)c1 | 0.265 |
| Fosaprepitant | C[C@@H](O[C@H]1OCCN(Cc2nc(=O)n(P(=O)(O)O)[nH]2)[C@H]1c1ccc(F)cc1)c1cc(C(F)(F)F)cc(C(F)(F)F)c1 | 0.186 |
| Leucovorin | Nc1nc2c(c(=O)[nH]1)N(C=O)C(CNc1ccc(C(=O)N[C@@H](CCC(=O)O)C(=O)O)cc1)CN2 | 0.153 |
| Chlordiazepoxide | CNC1=Nc2ccc(Cl)cc2C(c2ccccc2)=[N+]([O-])C1 | 0.148 |
| Salsalate | O=C(Oc1ccccc1C(=O)O)c1ccccc1O | 0.129 |
| Indacaterol | CCc1cc2c(cc1CC)CC(NC[C@H](O)c1ccc(O)c3[nH]c(=O)ccc13)C2 | 0.099 |
| Lodoxamide | N#Cc1cc(NC(=O)C(=O)O)c(Cl)c(NC(=O)C(=O)O)c1 | 0.080 |
| Lenalidomide | Nc1cccc2c1CN(C1CCC(=O)NC1=O)C2=O | 0.067 |
| Lindane | Cl[C@H]1[C@H](Cl)[C@@H](Cl)[C@@H](Cl)[C@H](Cl)[C@H]1Cl | 0.067 |
| Marimastat | CNC(=O)[C@@H](NC(=O)[C@H](CC(C)C)[C@H](O)C(=O)NO)C(C)(C)C | 0.058 |
| Deferasirox | O=C(O)c1ccc(-n2nc(-c3ccccc3O)nc2-c2ccccc2O)cc1 | 0.047 |
| Fluorescein | O=C1OC2(c3ccc(O)cc3Oc3cc(O)ccc32)c2ccccc21 | 0.041 |
| Dalfopristin | CCN(CC)CCS(=O)(=O)[C@@H]1CCN2C(=O)c3coc(n3)CC(=O)C[C@H](O)/C=C(C)/C=C/CNC(=O)/C=C/[C@@H](C)[C@@H](C(C)C)OC(=O)[C@@H]12 | 0.039 |
| Ademetionine | C[S+](CC[C@H](N)C(=O)O)C[C@H]1O[C@@H](n2cnc3c(N)ncnc32)[C@H](O)[C@@H]1O | 0.030 |
| Delafloxacin | Nc1nc(-n2cc(C(=O)O)c(=O)c3cc(F)c(N4CC(O)C4)c(Cl)c32)c(F)cc1F | 0.029 |
| Sulfamoxole | Cc1nc(NS(=O)(=O)c2ccc(N)cc2)oc1C | 0.029 |
| Epirubicin | COc1cccc2c1C(=O)c1c(O)c3c(c(O)c1C2=O)C[C@@](O)(C(=O)CO)C[C@@H]3O[C@H]1C[C@H](N)[C@@H](O)[C@H](C)O1 | 0.025 |
| Viomycin | N=C1N[C@@H](O)C[C@H]([C@@H]2NC(=O)/C(=C/NC(N)=O)NC(=O)[C@H](CO)NC(=O)[C@H](CO)NC(=O)[C@@H](NC(=O)C[C@@H](N)CCCN)CNC2=O)N1 | 0.024 |
| Telotristat | Cc1ccn(-c2cc(Cl)ccc2[C@@H](Oc2cc(-c3ccc(C[C@H](N)C(=O)O)cc3)nc(N)n2)C(F)(F)F)n1 | 0.023 |
| Ergocalciferol | C=C1CC[C@H](O)C/C1=C/C=C1\CCC[C@@]2(C)[C@H]1CC[C@@H]2[C@H](C)/C=C/[C@H](C)C(C)C | 0.018 |
| Entecavir | C=C1[C@@H](n2cnc3c(=O)nc(N)[nH]c32)C[C@H](O)[C@H]1CO | 0.017 |
| Alvimopan | C[C@H]1CN(C[C@H](Cc2ccccc2)C(=O)NCC(=O)O)CC[C@@]1(C)c1cccc(O)c1 | 0.016 |
| Aspartame | COC(=O)[C@H](Cc1ccccc1)NC(=O)[C@@H](N)CC(=O)O | 0.015 |
| Methylscopolamine bromide | C[N+]1(C)[C@H]2CC(OC(=O)[C@H](CO)c3ccccc3)C[C@H]1C1OC12 | 0.014 |
| Cefotaxime | CO/N=C(\C(=O)N[C@@H]1C(=O)N2C(C(=O)O)=C(COC(C)=O)CS[C@H]12)c1csc(N)n1 | 0.013 |
| Rolapitant | C[C@@H](OC[C@@]1(c2ccccc2)CC[C@]2(CCC(=O)N2)CN1)c1cc(C(F)(F)F)cc(C(F)(F)F)c1 | 0.012 |
| Ceftizoxime | CO/N=C(\C(=O)N[C@@H]1C(=O)N2C(C(=O)O)=CCS[C@H]12)c1csc(N)n1 | 0.011 |
| Quazepam | Fc1ccccc1C1=NCC(=S)N(CC(F)(F)F)c2ccc(Cl)cc21 | 0.010 |
| Iopanoic acid | CCC(Cc1c(I)cc(I)c(N)c1I)C(=O)O | 0.010 |
| Capreomycin | NCCC[C@H](N)CC(=O)NC[C@@H]1NC(=O)[C@H](CO)NC(=O)[C@@H](N)CNC(=O)[C@H]([C@H]2CCN=C(N)N2)NC(=O)/C(=C\NC(N)=O)NC1=O | 0.010 |
| Prasugrel | CC(=O)Oc1cc2c(s1)CCN(C(C(=O)C1CC1)c1ccccc1F)C2 | 0.009 |
| Eliglustat | CCCCCCCC(O)=N[C@H](CN1CCCC1)[C@H](O)c1ccc2c(c1)OCCO2 | 0.008 |
| Talniflumate | O=C1OC(OC(=O)c2cccnc2Nc2cccc(C(F)(F)F)c2)c2ccccc21 | 0.008 |
| Conivaptan | Cc1nc2c([nH]1)CCN(C(=O)c1ccc(NC(=O)c3ccccc3-c3ccccc3)cc1)c1ccccc1-2 | 0.008 |
| Ceftaroline fosamil | CCO/N=C(\C(=O)N[C@@H]1C(=O)N2C(C(=O)O)=C(Sc3nc(-c4cc[n+](C)cc4)cs3)CS[C@H]12)c1nsc(NP(=O)(O)O)n1 | 0.008 |
| Cinolazepam | N#CCCN1C(=O)C(O)N=C(c2ccccc2F)c2cc(Cl)ccc21 | 0.007 |
| Cariprazine | CN(C)C(=O)N[C@H]1CC[C@H](CCN2CCN(c3cccc(Cl)c3Cl)CC2)CC1 | 0.006 |
| Oxazepam | O=C1Nc2ccc(Cl)cc2C(c2ccccc2)=NC1O | 0.006 |
| Canagliflozin | Cc1ccc([C@@H]2O[C@H](CO)[C@@H](O)[C@H](O)[C@H]2O)cc1Cc1ccc(-c2ccc(F)cc2)s1 | 0.005 |
| Pioglitazone | CCc1ccc(CCOc2ccc(CC3SC(=O)NC3=O)cc2)nc1 | 0.005 |
| Nicergoline | CO[C@]12C[C@@H](COC(=O)c3cncc(Br)c3)CN(C)[C@@H]1Cc1cn(C)c3cccc2c13 | 0.005 |
| Tasosartan | Cc1nc(C)c2c(n1)N(Cc1ccc(-c3ccccc3-c3nn[nH]n3)cc1)C(=O)CC2 | 0.005 |
| Chloramphenicol | O=C(N[C@H](CO)[C@H](O)c1ccc([N+](=O)[O-])cc1)C(Cl)Cl | 0.005 |
| Lisdexamfetamine | C[C@@H](Cc1ccccc1)NC(=O)[C@@H](N)CCCCN | 0.005 |
| Elvitegravir | COc1cc2c(cc1Cc1cccc(Cl)c1F)c(=O)c(C(=O)O)cn2[C@H](CO)C(C)C | 0.005 |
| Atovaquone | O=C1C(O)=C([C@H]2CC[C@H](c3ccc(Cl)cc3)CC2)C(=O)c2ccccc21 | 0.005 |
| Adefovir Dipivoxil | CC(C)(C)C(=O)OCOP(=O)(COCCn1cnc2c(N)ncnc21)OCOC(=O)C(C)(C)C | 0.005 |
| Besifloxacin | N[C@@H]1CCCCN(c2c(F)cc3c(=O)c(C(=O)O)cn(C4CC4)c3c2Cl)C1 | 0.004 |
| Ceftazidime | CC(C)(O/N=C(\C(=O)N[C@@H]1C(=O)N2C(C(=O)O)=C(C[n+]3ccccc3)CS[C@H]12)c1csc(N)n1)C(=O)O | 0.004 |
| Pitavastatin | O=C(O)C[C@H](O)C[C@H](O)/C=C/c1c(C2CC2)nc2ccccc2c1-c1ccc(F)cc1 | 0.004 |
| Sulfadoxine | COc1ncnc(NS(=O)(=O)c2ccc(N)cc2)c1OC | 0.004 |
| Ceftriaxone | CO/N=C(\C(=O)N[C@@H]1C(=O)N2C(C(=O)O)=C(CSc3nc(=O)c(=O)[nH]n3C)CS[C@H]12)c1csc(N)n1 | 0.004 |

**REFERENCES**

1. Howard J, Ruder S (2018) Universal Language Model Fine-tuning for Text Classification. Proceedings of the 56th Annual Meeting of the Association for Computational Linguistics (Volume 1: Long Papers). https://doi.org/10.18653/v1/p18-1031

2. Xue D, Gong Y, Yang Z, Chuai G, Qu S, Shen A, Yu J, Liu Q (2019) Advances and challenges in deep generative models for de novo molecule generation. Wiley Interdisciplinary Reviews: Computational Molecular Science 9:e1395

3. Walters WP, Murcko M (2020) Assessing the impact of generative AI on medicinal chemistry. Nat Biotechnol 38:143–145

4. Brown TB, Mann B, Ryder N, et al (2020) Language Models are Few-Shot Learners. arXiv [cs.CL]

5. Makino T, Liao H, Assael Y, Shillingford B, Garcia B, Braga O, Siohan O (2019) Recurrent Neural Network Transducer for Audio-Visual Speech Recognition. In: 2019 IEEE Automatic Speech Recognition and Understanding Workshop (ASRU). pp 905–912

6. Güera D, Delp EJ (2018) Deepfake Video Detection Using Recurrent Neural Networks. In: 2018 15th IEEE International Conference on Advanced Video and Signal Based Surveillance (AVSS). pp 1–6

7. Segler MHS, Kogej T, Tyrchan C, Waller MP (2018) Generating focused molecule libraries for drug discovery with recurrent neural networks. ACS Central Science 4:120–131

8. Grisoni F, Moret M, Lingwood R, Schneider G (2020) Bidirectional Molecule Generation with Recurrent Neural Networks. J Chem Inf Model 60:1175–1183

9. Hochreiter S, Schmidhuber J (1997) Long short-term memory. Neural Comput 9:1735–1780

10. Gupta A, Müller AT, Huisman BJH, Fuchs JA, Schneider P, Schneider G (2018) Generative Recurrent Networks for De Novo Drug Design. Mol Inform. https://doi.org/10.1002/minf.201700111

11. Moret M, Friedrich L, Grisoni F, Merk D, Schneider G (2020) Generative molecular design in low data regimes. Nature Machine Intelligence 2:171–180

12. Bjerrum EJ, Threlfall R (2017) Molecular Generation with Recurrent Neural Networks (RNNs).

13. Méndez-Lucio O, Baillif B, Clevert D-A, Rouquié D, Wichard J (2020) De novo generation of hit-like molecules from gene expression signatures using artificial intelligence. Nat Commun 11:10

14. Olivecrona M, Blaschke T, Engkvist O, Chen H (2017) Molecular de-novo design through deep reinforcement learning. J Cheminform 9:48

15. Popova M, Isayev O, Tropsha A (2018) Deep reinforcement learning for de novo drug design. Science Advances 4:eaap7885

16. Zhavoronkov A, Zagribelnyy B, Zhebrak A, et al (2020) Potential non-covalent SARS-CoV-2 3C-like protease inhibitors designed using generative deep learning approaches and reviewed by human medicinal chemist in virtual reality.

17. Blaschke T, Arús-Pous J, Chen H, Margreitter C, Tyrchan C, Engkvist O, Papadopoulos K, Patronov A (2020) REINVENT 2.0--an AI tool for de novo drug design.

18. Zhou Z, Kearnes S, Li L, Zare RN, Riley P (2019) Optimization of Molecules via Deep Reinforcement Learning. Sci Rep 9:10752

19. Ståhl N, Falkman G, Karlsson A, Mathiason G, Boström J (2019) Deep Reinforcement Learning for Multiparameter Optimization in de novo Drug Design. https://doi.org/10.1021/acs.jcim.9b00325

20. Brown N, Fiscato M, Segler MHS, Vaucher AC (2019) GuacaMol: Benchmarking Models for de Novo Molecular Design. J Chem Inf Model 59:1096–1108
